# Molecular variation across populations of a widespread North American firefly, *Photinus pyralis*, reveals that coding changes do not underlie flash color variation or associated visual sensitivity

**DOI:** 10.1186/s12862-018-1251-9

**Published:** 2018-08-31

**Authors:** Sarah E. Lower, Kathrin F. Stanger-Hall, David W. Hall

**Affiliations:** 1 Department of Genetics, University of Georgia, Athens, GA 30602 USA; 2 Department of Plant Biology, University of Georgia, Athens, GA 30602 USA; 3 Present address: Department of Biology, Bucknell University, Lewisburg, PA 17837 USA

**Keywords:** Lampyridae, Bioluminescence, Fst outlier, Population genomics, Sensory drive

## Abstract

**Background:**

Genes underlying signal production and reception are expected to evolve to maximize signal detection in specific environments. Fireflies vary in their light signal color both within and between species, and thus provide an excellent system in which to study signal production and reception in the context of signaling environments. Differences in signal color have been hypothesized to be due to variation in the sequence of luciferase, the enzyme that catalyzes the light reaction. Similarly, differences in visual sensitivity, which are expected to match signal color, have been hypothesized to be due to variation in the sequence of opsins, the protein component of visual pigments. Here we investigated (1) whether sequence variation in luciferase correlates with variation in signal color and (2) whether sequence variation in opsins correlates with inferred matching visual sensitivity across populations of a widespread North American firefly species, *Photinus pyralis*. We further tested (3) whether selection has acted on these loci by examining their population-level differentiation relative to the distribution of differentiation derived from a genome-wide sample of loci generated by double-digest RADseq.

**Results:**

We found virtually no coding variation in luciferase or opsins. However, there was extreme divergence in non-coding variation in luciferase across populations relative to a panel of random genomic loci.

**Conclusions:**

The absence of protein variation at both loci challenges the paradigm that variation in signal color and visual sensitivity in fireflies is exclusively due to coding variation in luciferase and opsin genes. Instead, flash color variation within species must involve other mechanisms, such as abdominal pigmentation or regulation of light organ physiology. Evidence for selection at non-coding variation in luciferase suggests that selection is targeting luciferase regulation and may favor differ expression levels across populations.

**Electronic supplementary material:**

The online version of this article (10.1186/s12862-018-1251-9) contains supplementary material, which is available to authorized users.

## Background

Communication has been the subject of intense study since Darwin [1]. All communication systems involve the transmission of a signal that carries information from a sender to a receiver (reviewed in [2]). Because transmission occurs through the environment, natural selection is expected to favor both signals and receptors that maximize signal detection in the context of ambient environmental conditions. As such, the evolution of any signaling system is affected (“driven”) by the characteristics of both the species and the biotic and abiotic environment. The “sensory drive” framework posits that signal production and reception are expected to evolve to maximize signal detection in the specific environment in which signals are displayed [3]. The effects of natural and sexual selection driving the evolution of sensory systems in particular directions should be detectable in the genes underlying signal production and detection. Such evidence for selection should be observable both across species and across populations within a species that inhabit different environments.

Fireflies, in the beetle family Lampyridae, are an established system for the study of mating signal evolution due to their conspicuous and variable light displays (e.g. [4–16]. Generally, in nocturnal species, flying males direct species-specific flash patterns to sedentary females in the vegetation [17]. These species-specific flash patterns are used for mate recognition and mate choice [18–23], but there is also variation in signal emission color, ranging from green (554 nm) to yellow/orange (579 nm) [4, 6, 7, 11, 16, 24, 25]. Across species, emission color correlates with the time of evening activity, with early-active (around sunset) species having yellower signals, which are hypothesized to have evolved to contrast with ambient green environmental light reflected off the vegetation, thereby facilitating signal detection [4, 16]. This “contrast hypothesis” holds within species as well—within early-active species, population-level variation in emission color is correlated with the habitat in which signals are displayed such that populations that signal in “closed”, forested environments have yellower signals [16]. While color varies across species and populations, a single individual is generally consistent from night to night, even when brought into the lab [16]. In addition, there is evidence that peak visual sensitivity of the eye matches peak emission color across species [4, 6, 7, 11], suggesting coevolution of vision and emission color at the species-level. This variation in signals, environments, and visual sensitivity provides a context for examining the molecular evolution of genes underlying signal production and reception as predicted by sensory drive.

Here, we undertook a candidate gene population genetics approach to test whether genes that underlie signal production and reception have evolved in the context of population-level variation in signaling environment and signal color. In fireflies, the primary genes underlying both light signal production (luciferase) and visual reception of the signal (opsins) are known. To produce a light signal, the enzyme luciferase interacts with its substrate, luciferin, in the presence of oxygen and ATP to release a photon of light (e.g. [26], reviewed in [27]. Previous studies, generally based on sequences from single individuals or single sampling locations, have shown that the amino acid sequence of luciferase is mostly conserved across firefly species, but varies at specific sites within the molecule [28–37]. Because luciferase is widely used as a luminescent marker in molecular studies, specific amino acid substitutions at these sites have been shown to be associated with in vitro changes in light color [28–37]. Thus, one favored paradigm predicts that signal color variation results directly from variation in the luciferase amino acid sequence [30]. Alternatively, color variation could arise from multiple expressed luciferases and/or other molecules that affect the morphological or physiological environment within the light organ. Fireflies do have two luciferase paralogs; however, in the species investigated to date, only one copy (*LUC1*) is expressed in the adult light organ and functions to produce flash signals [38–40].

To detect a light signal, visual pigments in the photoreceptors of the eye absorb photons of light, then transduce the signal to the optic nerve. Visual pigments are composed of two parts: a light-sensing vitamin A-derived chromophore and a signaling protein, opsin [41]. While opsins are mostly conserved, amino acid sequence variation at particular sites within the proteins, specifically substitutions at sites that interact with the chromophore, are known to affect visual sensitivity [42–44]. Fireflies have only two opsins, one that detects long-wavelength light (LW opsin) and one that detects ultraviolet wavelengths (UV opsin) [15, 45, 46]. Since there is no known UV component to the firefly light signal, flash signals are likely detected solely by the LW opsin [25], while UV opsin may be used for navigation through the environment [47], or in determining the onset of crepuscular activity [8, 9].

Here, we capitalize on documented variation in signal color across populations of a widespread North American species, *P. pyralis* [16], to investigate the evolution of luciferase and opsins with respect to signal color and environment. *P. pyralis* is a particularly good species in which to examine this because there are significant differences in signal color (mean wavelength at peak intensity) across populations, and the color range across populations spans ~ 60% of the entire color range of all measured firefly species. Applying the species paradigm, we hypothesized that natural selection on coding variation in adult-expressed luciferase and LW opsin also underlies these expansive differences in emission color among *P. pyralis* populations and their inferred matching visual sensitivities. We tested this hypothesis by determining whether within-species genetic variation in luciferase and LW opsin is correlated with variation in the color of emitted light across populations of *P. pyralis*. We predicted that nonsynonymous substitutions in the coding sequences of adult luciferase and LW opsin would be correlated with emission color. We further predicted that molecular variation in these genes would exhibit signatures of divergent selection across populations that differ in light color. Lastly, if there is evidence for selection causing divergence in luciferase and opsins across populations, we predicted that it would be driven by the light environment (habitat) in which signals are produced and received. Specifically, allele frequencies at selected loci were expected to correlate with differences in habitat. As there is no variation in the onset of crepuscular activity across *P. pyralis* populations, UV opsin amino acid sequence was not expected to be under selection and thus served as a control.

To test our hypotheses, we sequenced adult-expressed luciferase, LW opsin, and UV opsin across 12 *P. pyralis* populations exhibiting different peak emission colors and habitats. We tested for selection at specific sites within these loci by examining their pattern of molecular variation relative to a set of genome-wide single nucleotide polymorphisms (SNPs) generated from double-digest restriction-site associated DNA sequencing (ddRADseq [48];). We then examined population structure in *P. pyralis* using a set of neutrally-evolving ddRADseq SNPs and tested for correlations between genotype at luciferase or opsins and emission color or habitat while controlling for this population structure.

## Results

### Variation at signaling loci

**Fig. 1:**
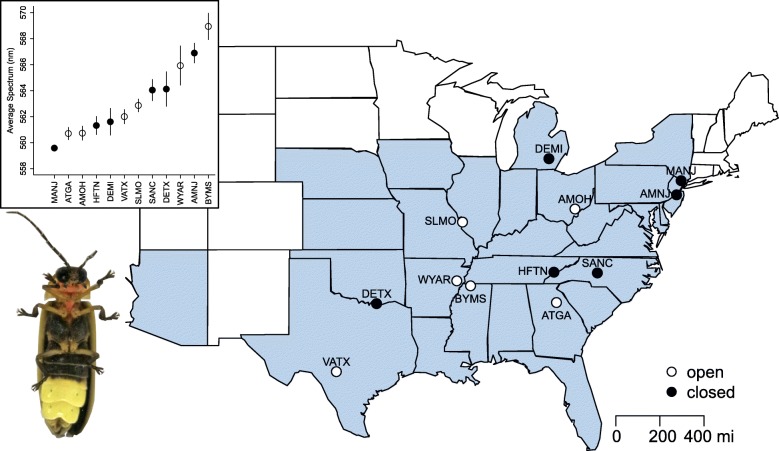
*Photinus pyralis* sampling sites. Individuals used for sequencing were collected from 12 locations during May–July of 2011–2014. Shading shows states that have at least one record of *P. pyralis* presence [16, 18]. Populations are labeled with a 4-letter location code and colored by habitat type. Inset (top left) is the distribution of male mean peak emission spectrum (+/− 1 SE) across the populations, ordered by increasing wavelength (from yellow to more orange). Sample sizes for emission measurements are shown above. Lower left shows the ventral aspect of an adult male. This map was generated using the maps package in R

To examine variation at signaling loci in the context of signaling environment, we Sanger sequenced the three signaling loci: adult luciferase, LW opsin, and UV opsin, of 191 individual males collected from 12 populations (15–16 individuals/population) across the *P. pyralis* North American geographical range. These populations differed in their mean peak emission color (558 to 568 nm) and habitat type (6 open field populations and 6 closed forest populations) ( [16]; Fig. 1; Additional File 1, Note 1). As predicted, all three loci showed sequence variation in coding regions (number of polymorphic sites in exons after removing singletons: LUC1:14, LW:19, UV:24). However, nonsynonymous variation that would result in a change in amino acid sequence was rare or absent (Table 1). Luciferase had a single nonsynonymous mutation, V182I, in exon 3 that was heterozygous in four individuals from Vanderpool, Texas (synonymous changes: 13, CDS length: 1650 bp). LW opsin had a single nonsynonymous mutation, A16V, in exon 1 that was heterozygous in four individuals, three from Dexter, Michigan (DEMI), and one from Byhalia, Mississippi (BYMS) (synonymous changes: 18, CDS length: 1134 bp). UV opsin had no nonsynonymous substitutions (synonymous changes: 26, CDS length: 1152 bp).

**Table 1:** Genetic diversity and gene flow estimates for adult luciferase (LUC1), and LW and UV opsin. Measures are given for the alignment of entire sequences (ALL) and only coding sequences (CDS). The number of individuals sequenced (N), number of variable sites (VS), number of synonymous (Syn) and nonsynonymous (NSyn) mutations within the coding sequence, the position of nonsynonymous mutations in the nucleotide alignment (Site), haplotype diversity (Hd +/− (SD)), nucleotide diversity (*π* +/− (SD)), Fst [84], and Tajima’s D [104] are given

Locus	Length (bp)	N	VS	Syn	NSyn	Site	Hd	*π*	Fst	Tajima’s D
LUC1: ALL	1966	190	21	13	1	667	0.69 (0.021)	0.00099 (0.00004)	0.61	−1.08
LUC1: CDS	1650	190	14	13	1	544	0.63 (0.018)	0.00080 (0.00003)	0.65	−0.91
LW: ALL	1459	191	24	18	1	98	0.79 (0.019)	0.00161 (0.00006)	0.20	−0.94
LW: CDS	1134	191	19	18	1	47	0.78 (0.019)	0.00200 (0.00007)	0.20	−0.55
UV: ALL	1440	191	41	26	0	N/A	0.91 (0.007)	0.00382 (0.00010)	0.31	−0.46
UV: CDS	1152	191	24	26	0	N/A	0.90 (0.007)	0.00351 (0.00009)	0.34	0.04

**Fig. 2:**
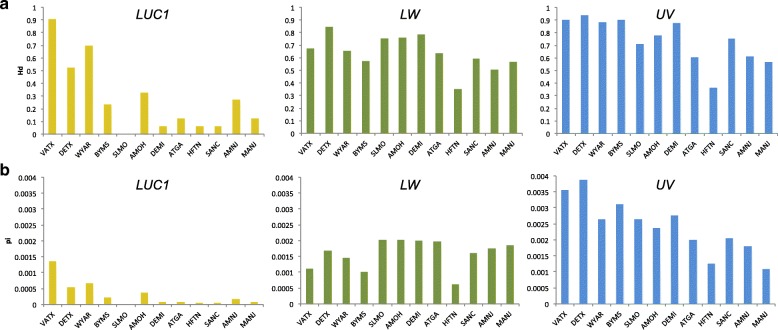
Genetic diversity statistics across populations at three loci involved in signal production/reception. On average, adult luciferase has lower genetic diversity than either LW or UV opsin across populations (given by 4-letter location code). Populations are ordered by Great Circle distance from the VATX population. Luciferase also exhibits a sharp decline in diversity with increasing distance from VATX, whereas the decline is more gradual in UV opsin and absent in LW opsin. (a) Haplotype diversity (Hd), (b) nucleotide diversity (π), adult luciferase (*LUC1*: gold), LW opsin (LW: green), UV opsin (UV: blue)

Genetic diversity differed among the three signaling loci and 12 populations. Luciferase had the lowest amount of diversity as measured by heterozygosity (Hd) and average pairwise nucleotide differences (π), but the highest amount of differentiation among populations (Fst) and a signature of excess low frequency polymorphisms (negative Tajima’s D) (Table 1). UV opsin had the highest diversity, lowest amount of differentiation, and most positive Tajima’s D. LW opsin was intermediate for diversity, differentiation, and Tajima’s D. Luciferase sequence diversity was highest in the most southern population (VATX), shown to be on a basal branch in a phylogenetic analysis of the mitochondrial *cytochrome oxidase I* (*COI*) locus (Additional File 1, Note 2), and decreased steeply with increasing distance. In contrast, the diversity of UV opsin declined more gradually with distance, and LW opsin diversity was maintained across populations (Fig. 2).

### Fst outliers

To test for selection and examine genetic differentiation at the three signaling loci relative to genome-wide genetic differentiation, we generated double-digest restriction-site associated DNA (ddRAD) libraries for 15 individuals from each of the 12 *P. pyralis* populations. Illumina sequencing of these libraries resulted in 190,648,449 PE75 reads. After quality trimming, cleaning contaminants, and discarding low-coverage libraries, we obtained data for 154 of the original 180 individuals with an average of 1,038,853 +/− 413,471 reads per specimen (11–15 individuals/population). Applying the Stacks pipeline with optimized parameters to the data resulted in 2019 variable loci across populations that were suitable for population analysis (i.e. not ascribed to mitochondrial DNA or prokaryotic contaminants). All loci were determined to be in Hardy Weinberg equilibrium within populations after Bonferroni correction for multiple testing.

To generate a set of neutral loci for use in population structure analyses we performed Fst outlier analysis on the 2019 RAD loci. Outliers were required to be in the top (diversifying) or bottom (balancing/purifying) 5% of Fst values across all 10 LOSITAN [49] and have a q-value < 0.01 across 2 BayeScan [50] runs (Additional File 1, Note 3) to be considered under selection. This analysis yielded 42 candidate loci under selection, 29 under diversifying and 13 under balancing or purifying selection, and 1977 neutral loci. None of the outlier RAD loci had a significant BLAST hit to a known gene. Neutral mean Fst across populations was estimated at 0.38 (LOSITAN) and 0.36 (BayeScan). The 1977 neutrally evolving loci identified by this analysis were retained for investigation of population structure.

**Fig. 3:**
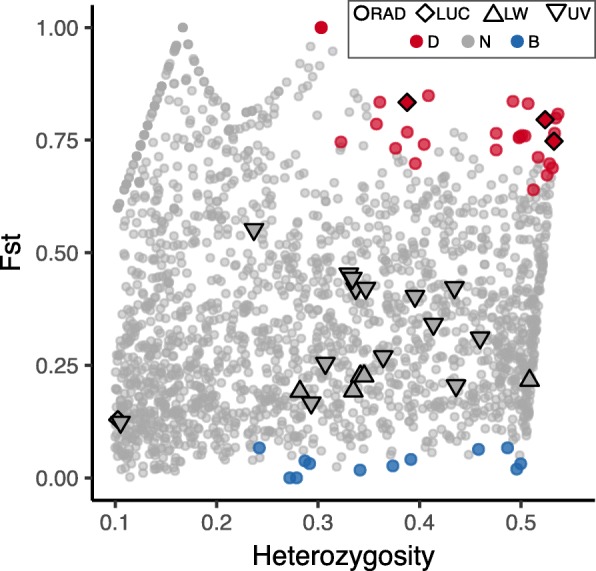
Fst outlier plot. Fst versus among-population heterozygosity for RAD loci (circles) plus SNPs from adult luciferase (LUC), LW opsin, and UV opsin (outlined symbols). Colors indicate candidate selection status: red – diversifying, gray – neutral, blue – balancing or purifying. Candidate selection status was determined from the strict consensus of 10 LOSITAN [49] runs and 2 Bayescan [50] runs. Only loci with heterozygosity above 0.1 are displayed (full figure given in Additional File 1, Note 3). The three luciferase SNPs with high heterozygosity (top right) are strong candidates for diversifying selection

To test signaling-related loci for evidence of selection, we combined the 2019 RAD loci and the variable SNPs from adult luciferase, LW opsin, and UV opsin, and then performed Fst outlier analysis using the same criteria on this combined dataset. This resulted in a slightly larger consensus set of 46 loci under selection (33 diversifying, 13 balancing or purifying) and 2058 neutral (Fig. 3). Loci under diversifying selection included the previously-identified 29 RAD loci, plus one new RAD locus, and 3 luciferase SNPs (sites 630, 723, 1780; numbered according to their position in Supplemental File 2a on Figshare). Sites 630 and 723 are 3rd positions within exon 3 of the luciferase gene, while site 1780 is in intron F, the penultimate intron of the gene. The other 18 variable luciferase SNPs had no evidence for extreme values of Fst, possibly because they had low levels of among-population heterozygosity (≤ 0.1). The single nonsynonymous mutation in luciferase was among these neutral SNPs. No SNPs from LW or UV opsin were identified as under selection in the consensus set of loci -- while LOSITAN identified two LW and two UV opsin SNPs as under balancing/purifying selection consistently across all 10 runs, these SNPs were not identified by BayeScan, and thus were excluded from the consensus set.

### Population structure

**Fig. 4:**
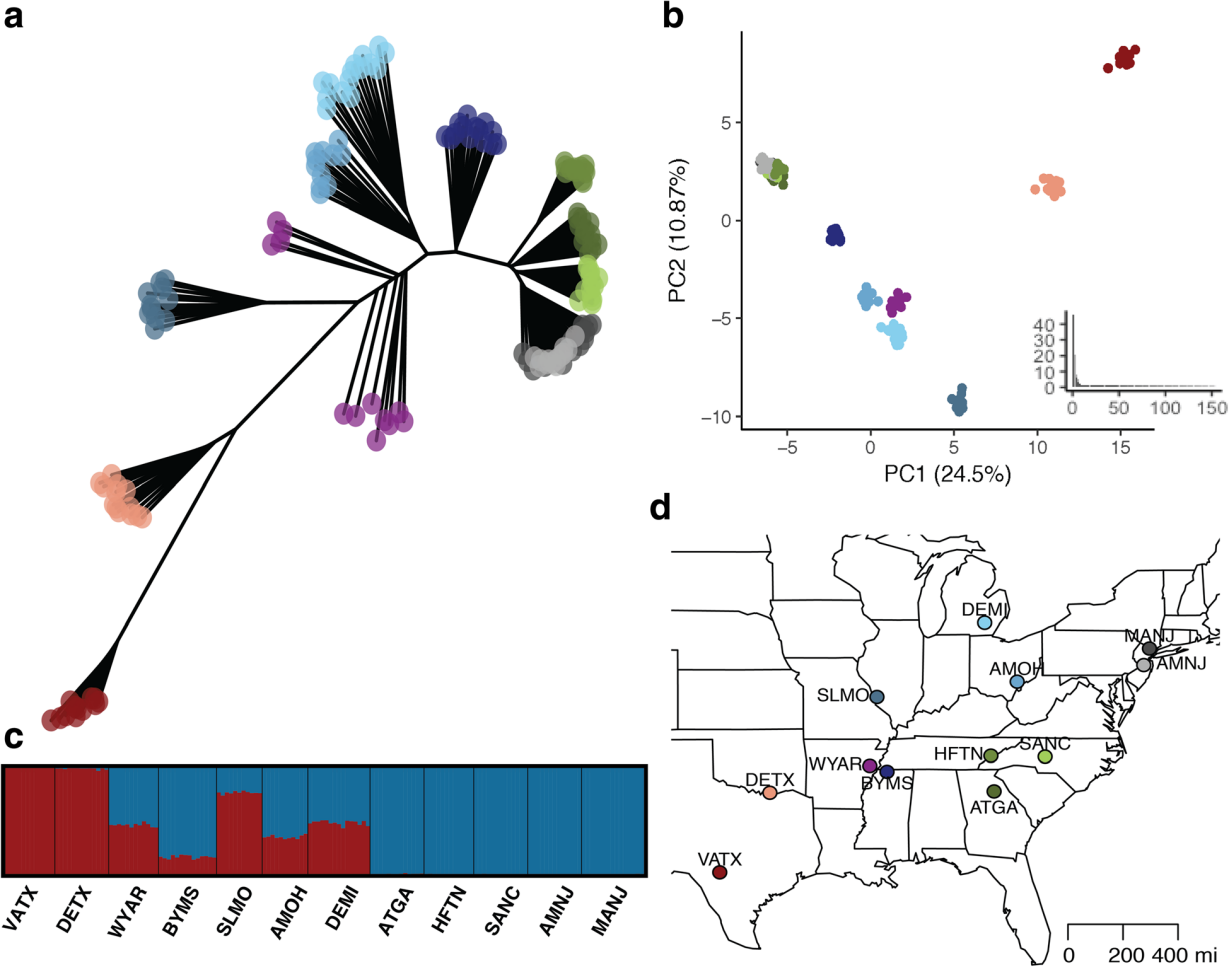
*Photinus pyralis* population structure. a) Neighbor-joining dendrogram of 154 *P. pyralis* from 12 populations. Unrooted dendrogram constructed from genetic distances between individuals [94] based on 1977 neutral SNPs. Individual specimens are labeled using colored circles that correspond to their population of origin. b) Principal components analysis of neutral SNPs: PC1 and PC2. Inset shows the eigenvalues of the retained components. Percent variance attributed to each component given in parentheses. Individual specimens are labeled using colored circles that correspond to their population of origin. c) STRUCTURE results with K = 2. Posterior probabilities of membership in each of 2 clusters across 154 individuals. Columns show stacked probabilities of membership for each individual. Dark lines separate individuals by population of origin, indicated by the locality code below. Populations are ordered left to right by Great Circle distance from VATX. The three clusters roughly correspond to two regions: South-West (red) and North-East (blue), with evidence for admixture between adjacent regions. d) Map showing the color key for sampled populations. This map was generated using the maps package in R

To place genetic variation at signaling loci in the context of biogeography, we investigated population structure and gene flow among the 12 *P. pyralis* populations. Genetic distance-based neighbor-joining dendrograms of neutral loci showed that specimens from each population were generally monophyletic, suggesting little gene flow between most populations, with the exception of two pairs of populations east of the Appalachians (Fig. 4a). The two populations from New Jersey (AMNJ and MANJ) were the geographically closest (96 km) population pair sampled and showed substantial overlap in the neighbor-joining tree, suggesting either contemporary gene flow between these two localities or recent separation with insufficient time for genetic differentiation. The topology also showed that Texas populations are genetically distinct from the other populations, which is supported by *COI* phylogenetic analysis (Additional File 1, Note 2). Populations East of the Appalachian Mountains were less genetically diverse, as evidenced by short branch lengths between populations ranging in latitude from Georgia to New Jersey. This pattern, of divergent Texas populations with less genetic differentiation among populations samples East of the Appalachians, was also supported by estimates of pairwise Fst among populations (Additional File 1, Note 4).

Principal Components Analysis (PCA) yielded two major components that together accounted for ~ 30% of the variance (Fig. 4b). PC1 (24.5%) roughly corresponded with latitude, while PC2 (10.87%) roughly corresponded with longitude, with the exception of the two divergent Texas populations. STRUCTURE results supported K = 2, determined by the Evanno method which finds the K that maximally improves the likelihood among successive tested K values, as the most likely number of genetic clusters in the dataset (Fig. 4c). The two clusters correspond to South-Western and North-Eastern groups, with evidence of admixture within mid-Western populations.

### Selection at the luciferase locus

**Fig. 5:**
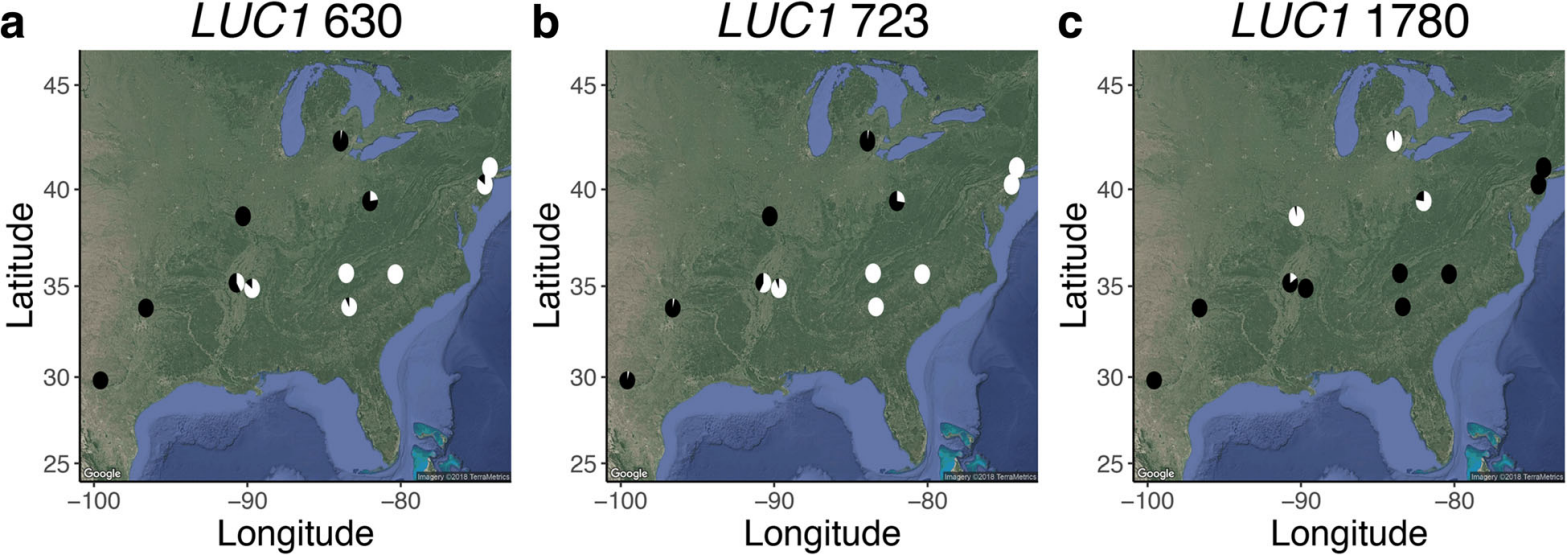
Distribution of allele frequencies across populations. Pie charts show allele frequencies across populations for the three adult luciferase SNPs with evidence for divergent selection: a) site 603, b) site 723, c) site 1780. Light and dark represent the frequencies of A and G alleles, respectively. Maps were generated using the R package ggmap [103] using the parameters: maptype = “satellite”, source = “google”

To understand how genetic variation at the three adult luciferase SNPs with evidence for divergent selection (sites 630, 723, 1780) was geographically distributed, we visualized allele frequencies at these loci across the populations sampled (Fig. 5). The genetic variation at these loci generally followed the expectation given the population structure analysis. Notably, populations were nearly fixed for a particular genotype at these three putatively selected luciferase SNPs (Fig. 5). In contrast, LW and UV opsin SNPs that were identified as candidates for balancing/purifying selection by LOSITAN (but not BayeScan; Additional File 1, Note 5) were generally not fixed within a population.

All 2019 RAD loci, combined with the signal locus SNPs, were tested for population-level associations in peak light emission color (wavelength of peak emission intensity) and habitat type (closed forest versus open field) using BayeScEnv [51]. This method uses a Bayesian framework to assess whether including a locus-specific environmental variable-associated component of Fst to model observed Fst at a locus has a higher posterior probability than models of Fst purely derived from demography or including other confounding locus-specific factors. Every individual in a given population was assigned the average wavelength and the habitat type for that population as measured in [16]. While 14 and 6 RAD SNPs were associated with wavelength and habitat, respectively (FDR < 0.05, consensus of 2 independent runs), none of the SNPs from signaling loci showed evidence for association with either emission wavelength or habitat type. Most of these SNPS (11/14 wavelength-associated, 3/6 habitat-associated) were also identified as under divergent selection in the initial screen for loci under selection using BayeScan. None of the RAD SNPs with evidence of association had a significant BLAST result.

## Discussion

While firefly signals have been the focus of much study, molecular approaches to investigating the genetic variation underlying this signal diversity have been few, likely limited by the dearth of DNA sequence data in this non-model insect system. Here, we took advantage of reference genome-free population genomic methods (ddRADseq, Fst outlier analysis) to investigate the genetic variation in luciferase and opsin genes among populations of a widely distributed North American firefly species, *P. pyralis*. These genes are instrumental for light signal production and light detection, respectively. We utilized 12 populations across the range (96 to ~ 3000 km apart) and population structure measures to test whether coding variation in signaling genes could explain differences in light emission color and the expected matching differences in visual sensitivity across different habitats.

### Population structure analysis suggests barriers to gene flow

*P. pyralis* is a widespread and abundant species. It is common across the Eastern U.S. and found in a variety of habitats, including urban and disturbed areas, and both males and females are able flyers. Therefore, we expected to observe relatively high levels of gene flow across the range (e.g. ladybird beetles: mean Fst = 0.039 [52]; pine beetle: Fst ranges from 0.02–0.1 across North American populations [53]), perhaps with more limited gene flow between populations at large geographic distances or across geographic boundaries that limit dispersal, such as the Appalachian mountains. Instead, the genome-wide SNP analysis showed a very high average Fst (0.38) among populations. Thus, it appears that *P. pyralis*, though widespread and abundant east of the Rockies, has limited gene flow among populations even though adults are able flyers. The adult stage is quite short (~ 2 weeks), during which males actively search for sedentary females, foregoing food consumption (but see [54]), and therefore adults may remain quite localized. In contrast to the mobile adult stage, *P. pyralis* has a long-lived terrestrial larval stage of up one to two years based on rearing data from the lab (personal observation), similar to other fireflies [55], and during this time the larvae likely also remain quite localized (on the order of 100 s of meters).

SNP analysis identified two large genetic clusters of *P. pyralis* populations, one in the South-West and one in the North-East. The division between these clusters roughly corresponds to the Appalachian Mountains, a known biogeographical break in a wide range of North American taxa (e.g [56–58]). Interestingly, the populations East of the Appalachians have evidence of short branches and/or more substantial gene flow (at the scale of 96–300 km) as compared to populations west of the Appalachians, a signature consistent with population expansion. Combined with evidence from the mitochondrial *COI* locus, the SNP data suggest a demographic scenario of range expansion from Texas northward, with rapid expansion up the eastern side of the Appalachians, though more rigorous demographic modeling efforts are required to disentangle likely demographic scenarios (e.g. glacial refugia, secondary contact, and divergence with gene flow).

It is critical to take population structure into account in evolutionary analyses. In addition, population structure has important implications for firefly conservation. *P. pyralis* was the original source for firefly luciferase used in molecular assays and millions of individuals were harvested each season for scientific use [59]. Following cloning of the *P. pyralis* luciferase in the 1980s [60], luciferase can now be obtained via firefly-free recombinant methods, but there remain anecdotal reports of continuing harvest. Models of population persistence in the face of substantial harvest, incorporating reductions in population growth rate and environmental change, suggest such levels of harvesting may not be sustainable [61]. The strong, localized population structure found in this study, where gene flow is very limited among populations, further suggests that migration is unlikely to be able to rescue threatened populations. Further, *P. pyralis* is a widespread, abundant, and robust species, more often associated with human agricultural activities compared to most other firefly species. The presence of high population structure in this species suggests that populations of other species may be even more genetically isolated. Thus, it is essential that firefly monitoring and conservation efforts take population structure into account.

### Luciferase, but not opsins, shows evidence of diversifying selection among populations

Contrary to our expectations based on prevailing hypotheses of the genetic basis of signal color and visual sensitivity and known differences in light emission color across populations, neither luciferase nor LW opsin showed a pattern of nonsynonymous changes in their coding sequence that was correlated with either their light emission wavelength or habitat type. Furthermore, the single low-frequency nonsynonymous mutation that we identified in luciferase (V182I) does not shift emission wavelength, though thermostability is altered [62]. The single low frequency nonsynonymous site in LW opsin is not predicted to affect visual sensitivity based on its predicted position outside of the chromophore binding pocket, based on homology models [15]. Previous work demonstrated that opsin amino acid variation did not directly match signal color variation across species [15]. We now add evidence that this also applies within a species, despite light color differences between populations. Opsins are expected to be mostly conserved, given the importance of visual reception for different functions (e.g. navigation, sensing time of day, light signal detection) across different life stages. However, this raises the question whether sensory drive occurs within a species, and if so, which alternative mechanism(s) underlie it.

Despite the lack of amino acid variation, three luciferase SNPs showed a strong signature of selection, based on their high Fst relative to the distribution generated from neutral genome-wide SNPs. This finding suggests that there has been selection-driven differentiation at a region linked to luciferase that has driven the observed differentiation of coding sequence of luciferase at silent sites. This region could be located either in a cis-regulatory sequence upstream of the luciferase coding region, or in another gene that is in strong linkage disequilibrium with luciferase and is the actual target of diversifying selection.

Not much is known about natural variation in luciferase regulation. The 5′ upstream region of *P. pyralis* luciferase has been examined in relation to related luciferase genes in other species, and a core promoter region has been identified [63, 64]. In addition, variation in 5′ and 3′ sequences flanking luciferase has been documented in the Asian firefly, *Luciola lateralis* [65], though the functional significance of sequence variation in flanking sequences remains to be investigated in *P. pyralis*. Interestingly, in *Lampyris noctiluca*, the European glowworm, an ancient transposon endonuclease domain is located 686 bp upstream of the start codon [64]. Transposons are often co-opted in gene regulation [66], though whether this particular insertion has functional significance, especially with respect to luciferase expression in natural populations, is unknown.

Luciferase regulation may be under selection because populations differ in optimal luciferase expression due to abiotic or biotic factors in the environment. For example, mean temperature differences among populations could affect turnover of luciferase, requiring altered expression to achieve optimal levels. In addition, females respond more readily to brighter flashes [67]. If the likelihood of finding a mate correlates with luciferase abundance, but the cost of expression differs across populations due to differences in water stress or limitations in larval diet, optimal expression could differ. Aggressive predatory *Photuris* fireflies that mimic light signals of their firefly prey species also cue in on light signals [68], so differences in predation levels across populations could also select for different optimal expression. Though these potential sources of selection are pure speculation at this point, they provide intriguing hypotheses for future work.

### Associations with emission wavelength and habitat

We detected no significant association between light emission color or habitat type and the luciferase and opsin SNP data across divergent populations of *P. pyralis*. Further, we found no amino acid variation that is likely to affect the color of the light produced by luciferase or the color sensitivity of opsins. While both molecules are expected to be conserved in sequence due to their essential functions in mate signal production and visual reception, the current paradigm for variation in light color, and matched variation in opsins, based on amino acid variation at specific sites within these conserved molecules, does not hold. Our data instead strongly suggest alternative mechanisms for the observed differences in light color across populations, such as physiological mechanisms in the light organ, morphological filtering of the color emitted by luciferase through screening pigments in the light organ [69], or through the impact of environmental factors such as pH and metal ions on light color (e.g. [70], reviewed in [71]). Similarly, instead of amino acid sequence variation in LW opsin, inferred differences in visual sensitivity may be fine-tuned by screening pigments in firefly eyes [7, 11, 72].

## Conclusion

Across widely-distributed *P. pyralis* populations, we found no evidence for selection on the amino acid sequence of UV and LW opsins, the two light-detecting proteins in firefly eyes. In contrast, the luciferase protein, which generates the light signal, exhibits large differences in allele frequency across populations for three SNPs, indicating positive selection for different luciferase haplotypes in different parts of the range. Surprisingly, none of these SNPs result in a change in the luciferase amino acid sequence, strongly suggesting that selection, rather than acting on the amino acid level, acts on variation that alters luciferase expression.

These findings are in stark contrast to the current paradigm that differences in flash signal color across firefly species are due to amino acid variation in luciferase. This study rejects the paradigm of coding variation in luciferase and opsins underlying signal color variation and visual reception, at least at the population-level. Instead, there is a strong signal of divergent selection in luciferase non-coding variation that points to luciferase regulation as a target of selection across populations, even though it is not correlated with habitat or signal color. This work provides a fascinating perspective on a long-established model for light color evolution, and it highlights the need for further study of mechanisms determining light color in natural populations.

## Methods

### Study system

*P. pyralis* is a widespread and abundant firefly species in the United States, ranging from Arizona to New York [18]. Adult light displays can be seen over fields and in woods from June to October, depending on locality. *P. pyralis* adult light color is yellow [4, 73], and peak emission color ranges 10 nm (558 nm: green/yellow to 568 nm: yellow/orange) across populations [16]. *P. pyralis* luciferase was the first to be cloned [60] and is widely used as a bioluminescent reporter in gene expression studies (reviewed in [27, 74]). In vitro, cloned *P. pyralis* luciferase emits at 560 nm [75–77].

### Sampling and DNA extraction

*P. pyralis* individuals were collected from 12 populations (locations) during the summer months of 2011–2014 (Fig. 1). For each population, the time of capture of the first specimen (activity start time), temperature at the beginning of activity, and habitat type (open field, closed forest, or mixed) were recorded, and emission spectra from at least five males measured [16]. Specimens were identified using both flash pattern (accounting for temperature) and morphology [18, 78], and were preserved in 95% ethanol for DNA analysis. At least one specimen per population was further confirmed molecularly by amplifying and sequencing 376 bp of *cytochrome oxidase I* (*COI*; primers HCO, LCO [13];) and comparing against an in-house database of over 400 *COI* sequences from 102 firefly taxa. This mitochondrial locus has been shown to be phylogenetically informative in *Photinus* fireflies [14]. All specimens are retained in the permanent KSH collection at the University of Georgia.

To capture molecular variation at loci involved in signal production/reception with respect to the rest of the genome, we sequenced the two opsins (LW and UV) and luciferase, and performed ddRADseq on individuals from these 12 populations (Fig. 1, Additional File 1, Note 1). The final dataset included 191 individuals: 15–16 *P. pyralis* individuals from each of six closed (forest) populations and six open (field) populations, that captured most of the range of male peak emission (560 nm–569 nm [16];; Fig. 1). Only male measurements and specimens were used since females are difficult to locate in the field. For all samples, genomic DNA was extracted from the thorax using a standard phenol chloroform isoamyl alcohol protocol with RNAse digestion (e.g. [79]).

### LUC1 and LW and UV opsin sequencing and analysis

Adult-expressed luciferase (*LUC1* [39];) and both firefly opsins (LW and UV [15];) were amplified using PCR and then bi-directionally sequenced on an Applied Biosystems 3730xl 96-capillary DNA Analyzer at the Georgia Genomics Facility (Athens, GA). To amplify each gene in its entirety, forward and reverse PCR primers were designed using Primer3 [80] from flanking sequences identified in *P. pyralis* transcriptome and genome sequences [15]. Both custom species-specific and previously published internal primers were then used to sequence each amplicon. Full-length genomic sequences, from start to stop codon, were obtained for each locus. All primer sequences and PCR cycling conditions are given in Additional File 1, Table S3. The luciferase gene from one individual could not be amplified in its entirety and so this individual was excluded from luciferase genetic variation analysis.

Sequences were assembled in Geneious R7 (Biomatters Ltd.) and manually inspected for errors and heterozygous sites. Full-length contigs were aligned using Muscle [81] in Geneious and annotated for exon-intron boundaries using coding sequences obtained from transcriptomes or downloaded from Genbank (LW and UV opsins [15]:; *LUC1*: M15077 [63],). Singletons and gaps in introns due to indels were removed prior to downstream analysis. Alignments were phased in DNAsp v5 ( [82]; 10,000 iterations, burnin of 10%, 2 independent runs assessed for convergence) and then haplotype diversity (Hd [83],), pairwise nucleotide diversity (π [83],), Fst [84], and Tajima’s D [104] calculated for both the whole molecule and the coding sequence (Number of sequences per locus: *LUC1*, 190; LW, 191; UV, 191).

### ddRADseq library preparation, pooling, and sequencing

Sanger-sequenced individuals were assessed for genome-wide molecular variation using ddRAD sequencing [48, 85]. To allow for downstream optimization of RAD-locus assembly parameters [86], only 15 of the 16 Sanger-sequenced individuals from each population were randomly chosen for ddRAD sequencing and, from those 15, one was randomly chosen to generate an additional, technical replicate to use for optimization. In total, there were 16 libraries per population (15 individuals plus one technical replicate), resulting in 192 individual libraries across two 96-well plates. To decrease bias due to library preparation batch effects, the 16 libraries from each population were equally divided among plates and randomly assigned to wells: six replicate pairs were randomly assigned to wells within the same plate, while the other six were split between plates.

Library construction followed a 3RAD protocol [87]. 3RAD differs from ddRADseq [48] by using three restriction enzymes, two to digest genomic DNA and one to cut adapter dimers. This increases the efficiency of sequencing shared loci across specimens because adapter-dimers are eliminated rather than sequenced, resulting in higher sequencing coverage depth of desired genomic DNA fragments. In this study, genomic DNA from each specimen was digested with ClaI and BamHI, and MspI was used as the adapter-dimer cutter. Following digestion, unique combinations of internal barcode adapters were ligated onto cut fragments. Internal barcodes ranged in length from 6 to 9 nucleotides, thus ensuring appropriate library complexity for the Illumina sequencing platform. To reduce bias in the libraries due to differential adapter amplification in the subsequent PCR step, all samples on one plate were pooled, then divided into 3 aliquots, and each aliquot labeled with a unique combination of Illumina i5 and i7 adapters. After amplification, aliquots were pooled by plate, resulting in two final libraries composed of different individuals. To reduce each library to a reproducible portion of genome across specimens, these final libraries were size selected separately using a Caliper LabChip XT (PerkinElmer) at the Savannah River Ecology Lab (Aiken, SC). The average size of fragments in the final libraries was 550 bp +/− 12.5%. To reduce the effect of lane on sequencing output, the two size-selected samples were then pooled and run on 50% of 4 lanes of PE75 Illumina NextSeq at the Georgia Genomics Facility (Athens, GA).

### ddRADseq analysis

To generate a set of SNP-containing RAD loci that were sequenced across all individuals for use in downstream analyses, we identified reads from each sample in our pool using unique combinatorial barcodes, eliminated potential contaminant reads, and then grouped these into RAD loci bioinformatically. Samples were initially demultiplexed into their respective aliquot libraries (three from each of two plates) by outer Illumina adapters using bcl2fastq v2.16.0.10 (Illumina, Inc.). Subsequently, the reads for individual specimens were identified from each of these demultiplexed pools and trimmed for quality using process_radtags in Stacks v1.29 (parameters: -q –r –renz_1 mspI –renz_2 bamHI –t 63 [88],). Reads for each sample were concatenated across aliquot libraries and non-eukaryotic contaminants removed from each specimen using kraken (parameters: --paired –db minikraken_20141208 [89];). Attempts to avoid gut microbes by isolating DNA from thorax were generally successful (median: 0.02% reads identified as contaminants; range: 0.003–35%).

High-quality paired-end reads were concatenated and run through the Stacks pipeline using default parameters for initial exploration prior to parameter optimization. We identified 28/192 libraries (15%) that “failed”, meaning that they had data for less than 50% of the loci that were shared across 80% of the samples. All of these libraries had fewer than 100,000 reads and were excluded from downstream analysis. The failed libraries included 3 of the technical replicate samples, leaving 9/12 samples with replicates for optimization. Technical replicates were used to find the optimal Stacks parameters according to the procedure of [86](*m* = 3, *M* = 4, *n* = 3, *max_locus_stacks* = 3) with the default SNP calling model. In total, 154 samples were used in the final analysis—all the unique samples that passed our “failure” threshold, plus the “best” sample from each replicate pair (i.e. the one with the most reads).

Stacks output was analyzed using the populations module in Stacks v1.31. SNP loci included in the final analysis were required to be present in at least 80% of the individuals per population and have a minor allele frequency of at least 5%. Loci were annotated by comparing the consensus sequence for each locus to all nucleotide sequences in Genbank (blastn, evalue: 1e-4, ID: 90% [90];) and those with hits to mitochondrial or microbial sequences excluded from analysis. Each locus was tested for Hardy-Weinberg equilibrium prior to population structure and selection analyses using pegas [91] in R with Bonferroni correction to account for multiple testing.

### Fst outlier analysis

Loci with evidence for balancing and positive selection were identified using two methods: (i) the FDIST method [92] as implemented in the LOSITAN Selection Workbench [49] and (ii) BayeScan v2.1 [50]. LOSITAN estimates the background Fst distribution (i.e. for the likely neutral loci) and then identifies loci with Fst values falling in the tails of the distribution, i.e. more extreme than the 95% confidence interval (CI). Loci with Fst values larger or smaller than the Fst CI were considered to be under diversifying or balancing selection, respectively. Following best practices, loci in each category (diversifying, balancing, neutral) were determined from the consensus of 10 runs of 1,000,000 simulations each (options: “Neutral” mean Fst, Force mean Fst). To be considered in the diversifying or balancing selection categories, a locus must be present in that category across all 10 runs.

BayeScan decomposes Fst into that shared across all loci (population-specific Fst) and locus-specific Fst. Candidate loci under selection are identified as loci where locus-specific Fst needs to be invoked to explain diversity at that locus. BayeScan was run twice (parameters: -n 5000 -thin 10 -nbp 20 -pilot 5000 -burn 50,000 -pr_odds 100) and convergence of the two runs assessed using Gelman and Rubin’s diagnostic as implemented in the coda package [93] in R v3.3.2 (R Core Team 2008). Only loci with q-values less than 0.01 in both runs were considered as candidates under selection, with a negative or positive alpha value indicative of balancing/purifying or diversifying selection, respectively.

First, all RAD loci were run with both methods to develop a set of neutral loci used to investigate population structure. Loci with evidence for selection across both LOSITAN and Bayescan methods were excluded from further analysis with neutral markers. Then, all RAD loci and variable SNPs identified in luciferase, LW opsin, and UV opsin were combined and run to investigate selection at these three candidate loci.

### Population structure

When investigating candidate loci under selection, it is important to place their variation in the context of geographical population structure. Thus, using the set of neutral RAD loci, we examined population structure in several ways: (i) we constructed a neighbor-joining dendrogram of individuals using Nei’s distance [94] using the StAMPP v1.4 [95] and ape v5.0 [96] packages in R. (ii) We examined clustering of individuals by genotype using Principal Components Analysis (PCA) as implemented in adegenet v2.1 [97] in R and admixture models in STRUCTURE v2.3.4 [98]. All STRUCTURE analyses were run for 500,000 iterations with a burn-in of 50,000. Analyses were repeated 20 times for each K value (1–13). The CLUMPAK server [99] was used to first identify the best number of clusters using the Evanno method (ΔK [100];), then assess STRUCTURE replicates for each K using CLUMPP [101](parameters: LargeKGreedy, 2000 random inputs), and finally, visualize clusters for each K using Distruct [102]. (iii) We examined gene flow among populations by estimating pairwise Fst using StAMPP (parameters: nboots = 100, percent = 95, nclusters = 12).

To examine variation in candidate SNPs under selection with respect to population structure, geographical distributions of allele frequencies were visualized using ggmap v2.7 [103] in R.

### Testing for associations between genotype, spectra, and habitat

If SNPs in LUC, LW, and UV underlie phenotypic variation in spectra and inferred visual sensitivity that are associated with habitat, SNP genotype should be associated with these variables across populations. We tested for associations of all RAD and signaling loci SNPs with spectra and habitat using BayeScEnv v1.1 [51] with default parameters. Similarly to BayeScan, BayeScEnv identifies loci associated with environmental variables by decomposing Fst into that derived from demography (genome-wide population Fst) and locus-specific Fst. In this method, locus-specific Fst can be further broken down into that associated with the environmental variable or that associated with some other confounding variable such as differences in mutation rate or background selection. Loci for which incorporation of locus-specific environmental-associated Fst is required are considered as candidates for local adaptation to the environmental variable. This method accounts for population structure by allowing demography-derived Fst to vary among populations. Spectra data were mean-centered and normalized by the standard deviation prior to testing. Two BayeScEnv runs were performed per each explanatory variable and assessed for convergence using Gelman and Rubin’s diagnostic. Loci with FDR < 0.05 were considered associated with explanatory variables.

## Additional files

Additional file 1: For Lower et al. Contents: Notes 1–5. 1. Methods. 2. COI analysis. 3. Fst outlier analysis. 4. Gene flow among populations. 5. Additional signaling locus SNPs. 6. Files on Figshare. (PDF 6217 kb)

## Abbreviations

AMNJ: Amwell, New Jersey
ATP: adenosine triphosphate
BYMS: Byhalia, Mississippi
CDS: coding sequence
COI: cytochrome oxidase I
ddRADseq: double-digest restriction-site associated DNA sequencing
DEMI: Dexter, Michigan
DNA: deoxyribonucleic acid
FDR: False discovery rate
LUC1: adult-expressed luciferase
LW: long-wavelength
MANJ: Mahwah, New Jersey
P: genus abbreviation for Photinus
PCA: Principal components analysis
SNP: single nucleotide polymorphism
UV: ultra-violet
VATX: Vanderpool, Texas
