# Supplementary material for: Molecular variation across populations of a widespread North American firefly, Photinus pyralis, reveals that coding changes do not underlie flash color variation or associated visual sensitivity
Source: BMC Evol Biol. 2018 Aug 31;18:129. doi: 10.1186/s12862-018-1251-9 (PMC6119266; doi:10.1186/s12862-018-1251-9)
Supplement: Supplementary file 1 — For Lower et al. Contents: Notes 1–5. 1. Methods. 2. COI analysis. 3. Fst outlier analysis. 4. Gene flow among populations. 5. Additional signaling locus SNPs. 6. Files on Figshare. (PDF 6217 kb) [file 12862_2018_1251_MOESM1_ESM.pdf]

TABLE OF CONTENTS

Supplemental Information: Note 1. Supplemental Methods
Table S1. 12 populations selected for sequencing
Table S2. Specimens in each dataset
Table S3. Primers and PCR cycling conditions

Supplemental Information: Note 2. COI analysis
Table S4. Specimens used in COI analysis
Figure S1. *Photinus pyralis* from Eastern states are derived from Western states
Figure S2. Expanded COI phylogeny

Supplemental Information: Note 3. Fst outlier analysis
Figure S3. Consensus set of Fst outliers without signaling loci
Figure S4. Consensus set of Fst outliers with signaling loci
Figure S5. Neighbor-joining phylogenies of selected loci

Supplemental Information: Note 4. Gene flow among populations
Figure S6. Pairwise Fst among populations
Table S4. Fst bootstrap results

Supplemental Information: Note 5. Additional signaling locus SNPs
Figure S7. Candidate signaling-related loci SNPs under balancing/purifying selection

Supplemental Information: Note 6. Files on Figshare

References

Supplemental Information: Note 1. Supplemental Methods

Table S1. Populations selected for sequencing

The twelve populations selected for investigation span the range of habitats and wavelengths measured for *Photinus pyralis* males (Hall, Sander *et al.* 2016). The four-letter population code (Code), collection date (Date), mean male peak emission wavelength (Spectra), standard error of male peak emission wavelength (SE), number of males measured for spectra (N), and habitat type (O: open, C: closed) are given for each population.

Code	Locality	Date	N	Spectra	SE	Habitat
AMNJ	Amwell, NJ	June 2013	5	566.89	0.76	C
AMOH	Amesville, OH	June 2012	4	560.73	0.55	O
ATGA	Athens, GA	May, July 2011-14	2	560.70	0.48	O
BYMS	Byhalia, MS	June 2012	6	565.83	0.46	O
DEMI	Dexter, MI	July 2013	5	561.60	1.03	C
DETX	Denison, TX	May 2013	6	564.12	1.34	C
HFTN	Hickory Flats Branch, TN	June 2012	4	561.32	0.70	C
MANJ	Mahwah, NJ	July 2012	6	559.59	0.10	C
SANC	Salisbury, NC	June 2013	6	564.04	0.82	C
SLMO	St. Louis, MO	June 2012	8	562.86	0.50	O
VATX	Vanderpool, TX	May 2013	8	562.00	0.55	O
WYAR	Wynne, AR	June 2013	5	565.92	1.51	O

Table S2. Specimens and Genbank information

Unique identifier in the Stanger-Hall collection at the University of Georgia (KSH), population (Code), Sanger-sequenced locus (LW: LW opsin, UV: UV opsin, LUC1: adult luciferase) presence (X) in the genetic diversity analysis, and presence (X) in the 3RAD dataset pre- and post- filtering for 192 *Photinus pyralis* males used in this study. One individual from each population was excluded at random from 3RAD library preparation to include technical replicates (3RADpre). Details on the localities where specimens were collected are available on Figshare: DOI 10.6084/m9.figshare.5771979.

KSH	Code	LW	UV	LUC1	3RADpre	3RADpost
11313	AMNJ	X	X	X	X	X
11314	AMNJ	X	X	X	X	X
11315	AMNJ	X	X	X	X	X
11316	AMNJ	X	X	X	X	
11317	AMNJ	X	X	X		
11318	AMNJ	X	X	X	X	X
11319	AMNJ	X	X	X	X	X
11320	AMNJ	X	X	X	X	X
11321	AMNJ	X	X	X	X	X
11322	AMNJ	X	X	X	X	X
11323	AMNJ	X	X	X	X	
11324	AMNJ	X	X	X	X	X
11325	AMNJ	X	X	X	X	X
11326	AMNJ	X	X	X	X	X
11327	AMNJ	X	X	X	X	X
11328	AMNJ	X	X	X	X	X
9335	AMOH	X	X	X	X	
9336	AMOH	X	X	X	X	X
9337	AMOH	X	X	X	X	X
9339	AMOH	X	X	X	X	X
9340	AMOH	X	X	X	X	X
9341	AMOH	X	X	X	X	X
9342	AMOH	X	X	X	X	X
9343	AMOH	X	X	X	X	
9344	AMOH	X	X	X	X	X
9345	AMOH	X	X	X	X	X
9347	AMOH	X	X	X	X	X
9350	AMOH	X	X	X	X	X
9351	AMOH	X	X	X	X	
9352	AMOH	X	X	X		
9353	AMOH	X	X	X	X	X
9354	AMOH	X	X	X	X	
8121	ATGA	X	X	X	X	X

8125	ATGA	X	X	X	X	X
8164	ATGA	X	X	X	X	X
8165	ATGA	X	X	X		
8174	ATGA	X	X	X	X	X
8618	ATGA	X	X	X	X	X
8622	ATGA	X	X	X	X	X
8871	ATGA	X	X	X	X	X
8872	ATGA	X	X	X	X	X
8880	ATGA	X	X	X	X	X
8881	ATGA	X	X	X	X	X
8882	ATGA	X	X	X	X	
8883	ATGA	X	X	X	X	
11496	ATGA	X	X	X	X	X
11497	ATGA	X	X	X	X	X
11499	ATGA	X	X	X	X	X
8963	BYMS	X	X	X		
8964	BYMS	X	X	X	X	X
8965	BYMS	X	X	X	X	X
8966	BYMS	X	X	X	X	X
8967	BYMS	X	X	X	X	X
8969	BYMS	X	X	X	X	X
8970	BYMS	X	X	X	X	X
8971	BYMS	X	X	X	X	X
8972	BYMS	X	X	X	X	X
8973	BYMS	X	X	X	X	
8974	BYMS	X	X	X	X	X
8976	BYMS	X	X	X	X	X
8977	BYMS	X	X	X	X	X
8978	BYMS	X	X	X	X	X
8979	BYMS	X	X	X	X	X
8981	BYMS	X	X	X	X	X
11451	DEMI	X	X	X	X	X
11452	DEMI	X	X	X	X	X
11454	DEMI	X	X	X	X	X
11455	DEMI	X	X	X	X	X
11456	DEMI	X	X	X		
11457	DEMI	X	X	X	X	X
11458	DEMI	X	X	X	X	X
11459	DEMI	X	X	X	X	X
11460	DEMI	X	X	X	X	X
11461	DEMI	X	X	X	X	X
11462	DEMI	X	X	X	X	X

11463	DEMI	X	X	X	X	X
11464	DEMI	X	X	X	X	X
11465	DEMI	X	X	X	X	X
11466	DEMI	X	X	X	X	X
11467	DEMI	X	X	X	X	X
11062	DETX	X	X	X	X	X
11063	DETX	X	X	X	X	X
11064	DETX	X	X	X	X	X
11065	DETX	X	X	X		
11066	DETX	X	X	X	X	X
11067	DETX	X	X	X	X	
11068	DETX	X	X	X	X	
11069	DETX	X	X	X	X	X
11070	DETX	X	X	X	X	X
11071	DETX	X	X	X	X	X
11072	DETX	X	X	X	X	X
11073	DETX	X	X	X	X	X
11075	DETX	X	X	X	X	X
11076	DETX	X	X	X	X	X
11077	DETX	X	X	X	X	X
11078	DETX	X	X	X	X	X
11533	HFTN	X	X	X	X	X
11534	HFTN	X	X	X	X	
11536	HFTN	X	X	X	X	X
11537	HFTN	X	X	X	X	X
11538	HFTN	X	X	X	X	X
11539	HFTN	X	X	X	X	
11541	HFTN	X	X	X	X	
11542	HFTN	X	X	X	X	X
11544	HFTN	X	X	X	X	X
11546	HFTN	X	X	X	X	X
11547	HFTN	X	X	X	X	X
11548	HFTN	X	X	X	X	X
11549	HFTN	X	X	X		
11550	HFTN	X	X	X	X	X
11552	HFTN	X	X	X	X	X
11553	HFTN	X	X	X	X	X
10686	MANJ	X	X	X	X	X
10690	MANJ	X	X	X	X	X
10691	MANJ	X	X	X	X	X
10692	MANJ	X	X	X		
10694	MANJ	X	X	X	X	X

10695	MANJ	X	X	X	X	X
10696	MANJ	X	X	X	X	X
10697	MANJ	X	X	X	X	X
10698	MANJ	X	X	X	X	X
10700	MANJ	X	X	X	X	X
10701	MANJ	X	X	X	X	X
10702	MANJ	X	X	X	X	X
10703	MANJ	X	X	X	X	X
10704	MANJ	X	X	X	X	X
10705	MANJ	X	X	X	X	X
10706	MANJ	X	X	X	X	X
11169	SANC	X	X	X	X	X
11170	SANC	X	X	X	X	X
11171	SANC	X	X	X	X	X
11172	SANC	X	X	X		
11173	SANC	X	X	X	X	X
11174	SANC	X	X	X	X	X
11175	SANC	X	X	X	X	X
11176	SANC	X	X	X	X	
11177	SANC	X	X	X	X	X
11179	SANC	X	X	X	X	X
11180	SANC	X	X	X	X	X
11181	SANC	X	X	X	X	X
11182	SANC	X	X	X	X	X
11183	SANC	X	X	X	X	
11185	SANC	X	X	X	X	X
11187	SANC	X	X	X	X	X
9036	SLMO	X	X	X	X	X
9037	SLMO	X	X	X	X	X
9038	SLMO	X	X	X	X	X
9039	SLMO	X	X	X	X	X
9040	SLMO	X	X	X	X	X
9041	SLMO	X	X	X	X	X
9042	SLMO	X	X	X	X	X
9043	SLMO	X	X	X	X	
9044	SLMO	X	X	X	X	X
9045	SLMO	X	X	X		
9046	SLMO	X	X	X	X	X
9048*	SLMO	N/A	N/A	N/A	N/A	N/A
9149	SLMO	X	X	X	X	X
9150	SLMO	X	X	X	X	
9151	SLMO	X	X	X	X	X

9152	SLMO	X	X	X	X	
11019	VATX	X	X	X	X	X
11020	VATX	X	X	X	X	
11021	VATX	X	X	X	X	X
11022	VATX	X	X	X	X	X
11023	VATX	X	X	X	X	X
11024	VATX	X	X	X		
11030	VATX	X	X	X	X	X
11035	VATX	X	X	X	X	X
11038	VATX	X	X	X	X	X
11039	VATX	X	X	X	X	X
11040	VATX	X	X	X	X	X
11041	VATX	X	X	X	X	
11042	VATX	X	X	X	X	X
11043	VATX	X	X	N/A**	X	
11044	VATX	X	X	X	X	X
11045	VATX	X	X	X	X	X
11080	WYAR	X	X	X	X	X
11081	WYAR	X	X	X	X	X
11084	WYAR	X	X	X	X	
11089	WYAR	X	X	X	X	X
11090	WYAR	X	X	X	X	X
11091	WYAR	X	X	X	X	X
11092	WYAR	X	X	X	X	
11093	WYAR	X	X	X	X	X
11094	WYAR	X	X	X	X	
11095	WYAR	X	X	X	X	X
11096	WYAR	X	X	X	X	X
11097	WYAR	X	X	X	X	X
11098	WYAR	X	X	X	X	
11099	WYAR	X	X	X	X	
11100	WYAR	X	X	X	X	X
11101	WYAR	X	X	X	X	X
Total		191	191	190	180	154

* Excluded from final analysis because mis-labeled (actually BYMS)
** Excluded from final analysis because of difficulty in PCR amplification and sequencing.

Table S3. Primers and PCR cycling conditions.

Long-wavelength (LW) and ultraviolet (UV) opsin, and adult luciferase (LUC1) were amplified from genomic DNA using touchdown PCR with external primers. The names of forward (F) and reverse (R) primers, their nucleotide sequences, and where they were first reported (source) are given.

A. External primers

Full-length loci were amplified from genomic DNA using external primers designed from flanking sequences. PCR conditions using Qiagen taq polymerase were: initial denaturation at 94°C for 3 minutes, then 3-step cycles of 94°C for 45 seconds, the appropriate annealing temperature for 1 minute, and 72°C for the appropriate extension time (Ext) given the length of the locus. There was a final extension at 72°C for 3 minutes. A touchdown protocol was used to increase specificity of the primers: annealing began at the initial annealing temperature (Ann) and was decreased by 1°C each cycle for the first 7 cycles, then maintained at 7°C below the Ann for the remaining 28 cycles (total=35 cycles).

Locus	Primer	Sequence	Source	Ann (°C)	Ext (min:sec)
LW	LoutF	CATGGTGGTCGTGTTAATG	This study	66	2:00
	LoutR	TAGCCTGCAAGGTTATATTTAG	This study		
UV	UVP_-113F	see source	Sander and Hall 2015	56	2:00
	Uphiz_R	see source	Sander and Hall 2015		
LUC1	pyrluc_beginF	GGAATTCCTTTGTGTTACATTCT	This study	62	2:30
	pluc_endR	AAAATTACCATTCATCAATTTGC	This study		

B. Internal primers

Both external and internal primers were used to sequence all loci bidirectionally at the Georgia Genomics Facility (Athens, GA).

Locus	Primer	Sequence	Source
LW	LWT2_329F	see source	Sander and Hall 2015
	LWT2_568R	see source	Sander and Hall 2015
	LWP_807F	see source	Sander and Hall 2015
	LWT2_1004R	see source	Sander and Hall 2015

UV	UVphmidH_F	see source	Sander and Hall 2015
	UVP_2352R	see source	Sander and Hall 2015
	UVP_997F	see source	Sander and Hall 2015
	UV_1134R	see source	Sander and Hall 2015
LUC1	pluc_m2F	GCGTTATTTATCGGAGTTGC	This study
	pluc_m1R	TAGGCTGCGAAATGTTCATA	This study
	pluc_m4F	TATGTAAACAATCCGGAAGC	This study
	pluc_m3R	TTCGTCCCAGTAAGCTATGTC	This study
	pluc_m3F	TATGTGGATTTCGAGTCGTC	This study
	pluc_m2R	AGGGATCGTAAAAACAGCTC	This study

C. Secondary primers

Secondary primers were used to amplify specific exons that were difficult to sequence due to insertions/deletions in flanking introns. Most specimens did not require the use of secondary primers.

Locus	Primer	Sequence	Source
LW	LWpy_956R	TACAAATAGTGGTAAAAAGTACACG	This study
	LWP_-102F	TGTGAAGGTACATTCACTTGCAG	This study
UV	Pnpy_ex5F	TCAGAATGGAACTCCAAAAG	This study
	Pnpy_ex4F	GGCCAAAAAGATGAATGTAG	This study
	UV_1425R	see source	Sander and Hall 2015
	UVP_1435R	see source	Sander and Hall 2015
	UV_1417R	GTCGCAGCCGGTTCGGTCG	This study
LUC1	749r_Photinus	CCAAAACCGTGATGGAATGGAAC	This study

D. Primer placement

1. LW opsin

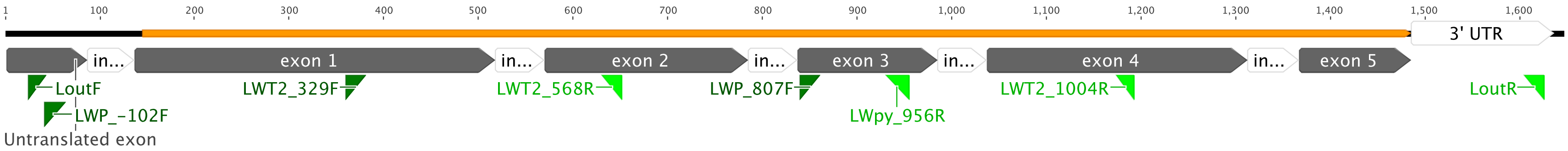

2. UV opsin

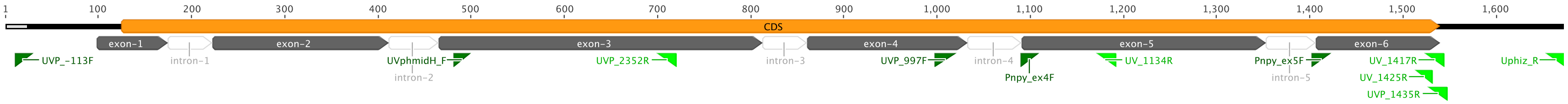

3. LUC1

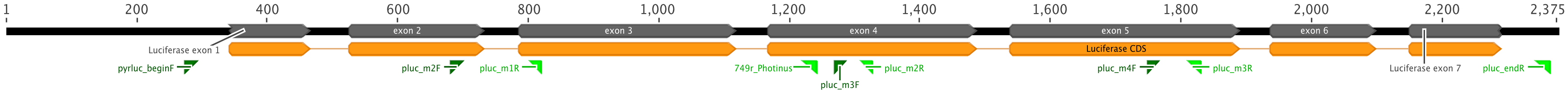

Supplemental Information: Note 2. *COI analysis*

Methods

To investigate population structure in *P. pyralis* we took advantage of the collection of over 400 mitochondrial *cytochrome oxidase I* (*COI*) partial sequences generated for fireflies by the Stanger-Hall lab over the past 15 years (primers: HCO-LCO; Stanger-Hall *et al.* 2007, Stanger-Hall & Lloyd 2015). In the lab these sequences are used for molecular verification of a specimen's species identity (Sander & Hall 2015, Hall, Sander *et al.* 2016). All *P. pyralis* sequences were extracted from the collection and combined with published *P. pyralis COI* sequences downloaded from Genbank for a total of 99 *P. pyralis* individuals collected from 45 localities across the Eastern United States during the summer from 1998-2013 (Table S4). A single *Photinus consisus*, three *Photinus carolinus* and one *Photuris quadrifulgens* served as outgroups (Stanger-Hall *et al.* 2007; Stanger-Hall & Lloyd 2015; Sander & Hall 2015).

Sequences were aligned using MUSCLE (Edgar 2004) in Geneious R7 (Biomatters Ltd.). The final alignment was trimmed to 555 bp of coding sequence with no gaps. jModeltest2 v.2.1.4 (Guindon & Gascuel 2003; Darriba *et al.* 2012) selected HKY + I + G as the best model of nucleotide substitution according to the AICc (corrected for small sample size; Posada 2008). Bayesian phylogenies were constructed in MrBayes v.3.2.1 (Ronquist *et al.* 2012) until the average standard deviation of split frequencies was below 0.01 (two independent runs, 5 million generations, ngammacat = 8, 25% burnin). Branches with support values below 50% were collapsed to yield the majority consensus tree.

Results

COI sequencing of 99 individuals from 45 localities resulted in 51 haplotypes. The resulting Bayesian phylogeny confirmed the monophyly of *P. pyralis* with high support. In addition, the topology showed that Western populations (Texas, Kansas, Arkansas and Mississippi) are basal to Eastern populations (remaining 12 states). Across the Eastern populations there was little phylogenetic resolution at the *COI* locus. The highest divergence occurred between specimens from Texas/Mississippi and New Jersey.

Table S4. Specimens used in COI analysis

Unique number in KSH collection (KSH), taxon (Species), 4-letter locality code (Location), year of capture, and Genbank accession for each specimen used to generate the COI phylogeny. Genus abbreviations: Pn.= *Photinus*, Pt. = *Photuris*, Pg. = *Pyropyga*. Locations: Amwell, NJ (AMNJ); Amesville, OH (AMOH); Ashdown, AR (ASAR); Athens, GA (ATGA); Athens, OH (ATOH); Bucks County, PA (BCPA); Belcamp/Harford, MD (BHMD); Bayles Road, Bloomington, IN (BRIN); Byhalia, MS (BYMS); Caldwell City, TX (CCTX); Chicago, IL (CGIL); Charleston, IL (CHIL); Charlottesville, VA (CHVA); Cranbury/Middlesex, NJ (CMNJ); Cookeville, TN (COTN); Denison, TX (DETX); Douglas/Lawrence, KS (DLKS); Doylestown, PA (DOPA); Easton, PA (EAPA); Eureka, MO (EUMO); Gonzales, TX (GOTX); Guadelupe River, TX (GRTX); Great Smoky Mountains National Park (GSMNP); Harrison, OH (HAOH); Hickory Flats Branch, TN (HFTN); Jackson County, TN (JCTN); Kutztown, PA (KUPA); Mahwah, NJ (MANJ); Montgomery Bell State Park, TN (MBTN); Montua, OH (MOOH); Moody, TX (MOTX); Nashville, TN (NATN); Ontelaunee Lake, PA (ONPA); Renfrew, PA (REPA); Salisbury, NC (SANC); State College, PA (SCPA); St. Louis, MO (SLMO); Vanderpool, TX (VATX); Whittington, IL (WHIL); Washington, D.C. (WSDC); Winston-Salem, NC (WSNC); Wynne, AR (WYAR). Outgroups (**OG**) are indicated in bold. 2 letter location abbreviations are state abbreviations.

KSH	Species	Location	Year	Genbank*
11273	*Pn. pyralis*	AMNJ	2013	
9330	*Pn. pyralis*	AMOH	2012	
9338	*Pn. pyralis*	AMOH	2012	
9349	*Pn. pyralis*	AMOH	2012	
11079	*Pn. pyralis*	ASAR	2013	
8025	*Pn. pyralis*	ATGA	2010	
8029	*Pn. pyralis*	ATGA	2010	
8175	*Pn. pyralis*	ATGA	2011	
8176	*Pn. pyralis*	ATGA	2011	
8819	*Pn. pyralis*	ATGA	2011	
8844	*Pn. pyralis*	ATGA	2011	
8846	*Pn. pyralis*	ATGA	2012	
8871	*Pn. pyralis*	ATGA	2012	
8872	*Pn. pyralis*	ATGA	2012	
8873	*Pn. pyralis*	ATGA	2012	
8880	*Pn. pyralis*	ATGA	2012	
8881	*Pn. pyralis*	ATGA	2012	
8882	*Pn. pyralis*	ATGA	2012	
8883	*Pn. pyralis*	ATGA	2012	
8892	*Pn. pyralis*	ATGA		
9311	*Pn. pyralis*	ATOH	2012	

9317	*Pn. pyralis*	ATOH	2012	
9318	*Pn. pyralis*	ATOH	2012	
11391	*Pn. pyralis*	BCPA	2013	
889	*Pn. pyralis*	BHMD	2001	KP121581.1[1]
9240	*Pn. pyralis*	BRIN	2012	
9028	*Pn. pyralis*	BYMS	2012	
975	*Pn. pyralis*	CCTX	2001	KP121582.1[1]
45	*Pn. pyralis*	CGIL	2001	
9160	*Pn. pyralis*	CHIL	2012	
10602	*Pn. pyralis*	CHVA	2012	
8470	*Pn. pyralis*	CHVA	2011	
8478	*Pn. pyralis*	CHVA	2011	
339	*Pn. pyralis*	CMNJ	2001	
8022	*Pn. pyralis*	COTN	2010	
11053	*Pn. pyralis*	DETX	2013	
289	*Pn. pyralis*	DLKS		
291	*Pn. pyralis*	DLKS		
292	*Pn. pyralis*	DLKS		
10802	*Pn. pyralis*	DOPA	2012	
10803	*Pn. pyralis*	DOPA	2012	
10804	*Pn. pyralis*	DOPA	2012	
8504	*Pn. pyralis*	DOPA	2011	
8509	*Pn. pyralis*	DOPA	2011	
8527	*Pn. pyralis*	DOPA	2011	
10825	*Pn. pyralis*	EAPA	2012	
9059	*Pn. pyralis*	EUMO	2012	
9077	*Pn. pyralis*	EUMO	2012	
10983	*Pn. pyralis*	GOTX	2013	
157	*Pn. pyralis*	GRTX	1998	
8365	*Pn. pyralis*	GSMNP	2011	
8366	*Pn. pyralis*	GSMNP	2011	
8369	*Pn. pyralis*	GSMNP	2011	KP121616.1[1]
9374	*Pn. pyralis*	HAOH	2012	
9384	*Pn. pyralis*	HAOH	2012	
9372	*Pn. pyralis*	HFTN	2012	
124	*Pn. pyralis*	IN	1999	
125	*Pn. pyralis*	IN	1999	
9020	*Pn. pyralis*	JCTN	2012	
9021	*Pn. pyralis*	JCTN	2012	
65	*Pn. pyralis*	KS		
9839	*Pn. pyralis*	KUPA	2012	
9852	*Pn. pyralis*	KUPA	2012	

10686	*Pn. pyralis*	MANJ	2012	
10690	*Pn. pyralis*	MANJ	2012	
10691	*Pn. pyralis*	MANJ	2012	
9025	*Pn. pyralis*	MBTN	2012	
17A	*Pn. pyralis*	MD		KP121624.1[1]
311	*Pn. pyralis*	MOOH	2001	
312	*Pn. pyralis*	MOOH	2001	
11046	*Pn. pyralis*	MOTX	2013	
11047	*Pn. pyralis*	MOTX	2013	
8852	*Pn. pyralis*	NATN	2012	
8857	*Pn. pyralis*	NATN	2012	
8862	*Pn. pyralis*	NATN	2012	
10045	*Pn. pyralis*	ONPA	2012	
9486	*Pn. pyralis*	REPA	2012	
9487	*Pn. pyralis*	REPA	2012	
11158	*Pn. pyralis*	SANC	2013	
63	*Pn. pyralis*	SCPA	2000	
102	*Pn. pyralis*	SLMO	1998	
9148	*Pn. pyralis*	SLMO	2012	
11021	*Pn. pyralis*	VATX	2013	
9109	*Pn. pyralis*	WHIL	2012	
72	*Pn. pyralis*	WI		
73	*Pn. pyralis*	WI		
8018	*Pn. pyralis*	WSDC	2010	
8019	*Pn. pyralis*	WSDC	2010	
322	*Pn. pyralis*	WSNC	2001	
323	*Pn. pyralis*	WSNC	2001	
324	*Pn. pyralis*	WSNC	2001	
325	*Pn. pyralis*	WSNC	2001	
11080	*Pn. pyralis*	WYAR	2013	
11526	*Pn. pyralis*			
11527	*Pn. pyralis*			
11528	*Pn. pyralis*			
11529	*Pn. pyralis*			
218	*Pn. pyralis*			
	Pn. pyralis			EU009313.1[2]
226	*Pn. concisus*	**OG**		KP121573.1[1]
9655	*Pn. carolinus*	**OG**		[3]
9660	*Pn. carolinus*	**OG**		[3]
9670	*Pn. carolinus*	**OG**		[3]
	Pt. quadrifulgens	**OG**		EU009310_1[2]
	Pg. decipiens	**OG**		KP121569.1[1]

*Blank indicates data generated in this study, Genbank accessions: MH759593 - MH759686
[1] Stanger-Hall and Lloyd 2015
[2] Stanger-Hall et al. 2007
[3] Faust et al. 2012

Figure S1. *Photinus pyralis* from Eastern states are derived from Western states This phylogeny, created from 555 bp of *COI* sequence, confirms the monophyly of *P. pyralis* and shows high support for Western populations being basal to Eastern populations. Abbreviations beneath regions are the states in which the specimens were collected. The cartoon depicts a large polytomy at the base of all Eastern specimens. Numbers next to nodes indicate Bayesian support values. Values over 0.7 are shown. Pcn: *Photinus concisus*, Pca: *Photinus carolinus*, Pgd: *Pyropyga decipiens*, Ptq: *Photuris quadrifulgens*. The full phylogeny is given in Figure S2.

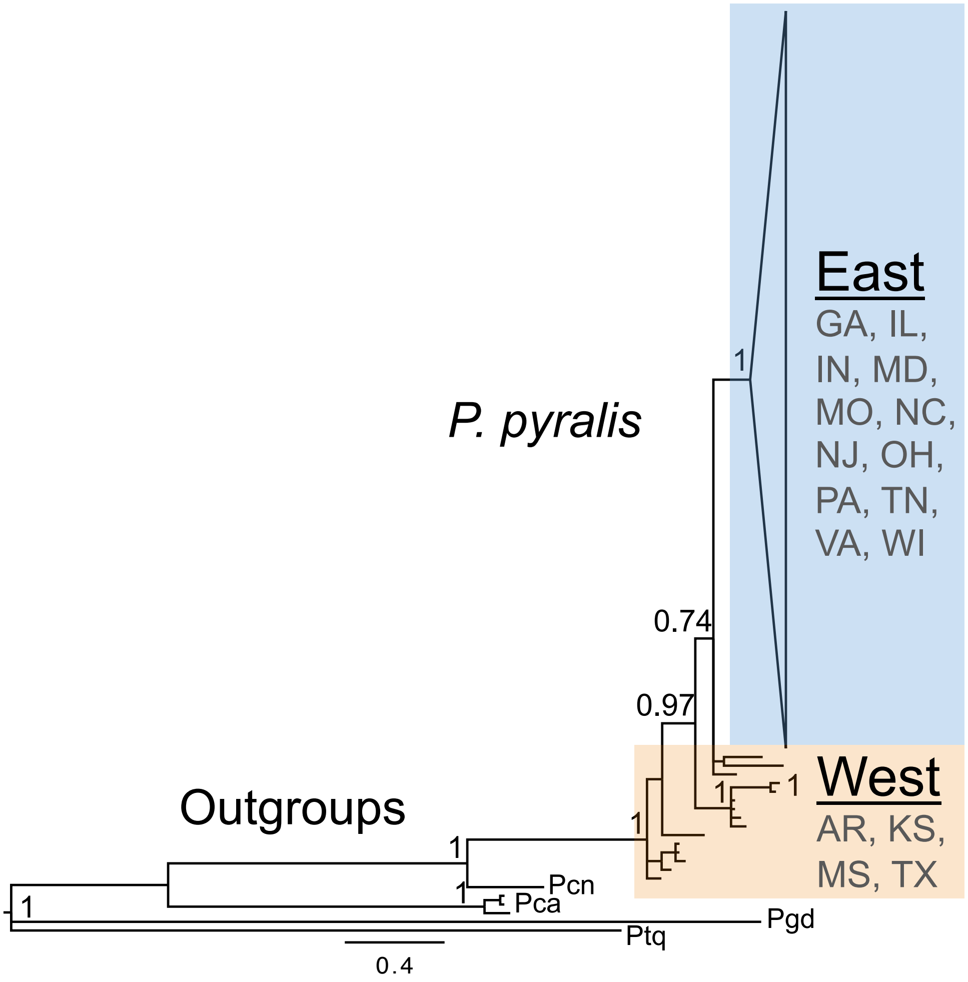

Figure S2. Expanded COI phylogeny

Phylogeny with individual specimen names indicated. Values at nodes are Bayesian support values. Pt = *Photuris*, Pg = *Pyropyga*, Py = *Photinus pyralis*, cn= *Photinus consicus*, ca = *Photinus carolinus*.

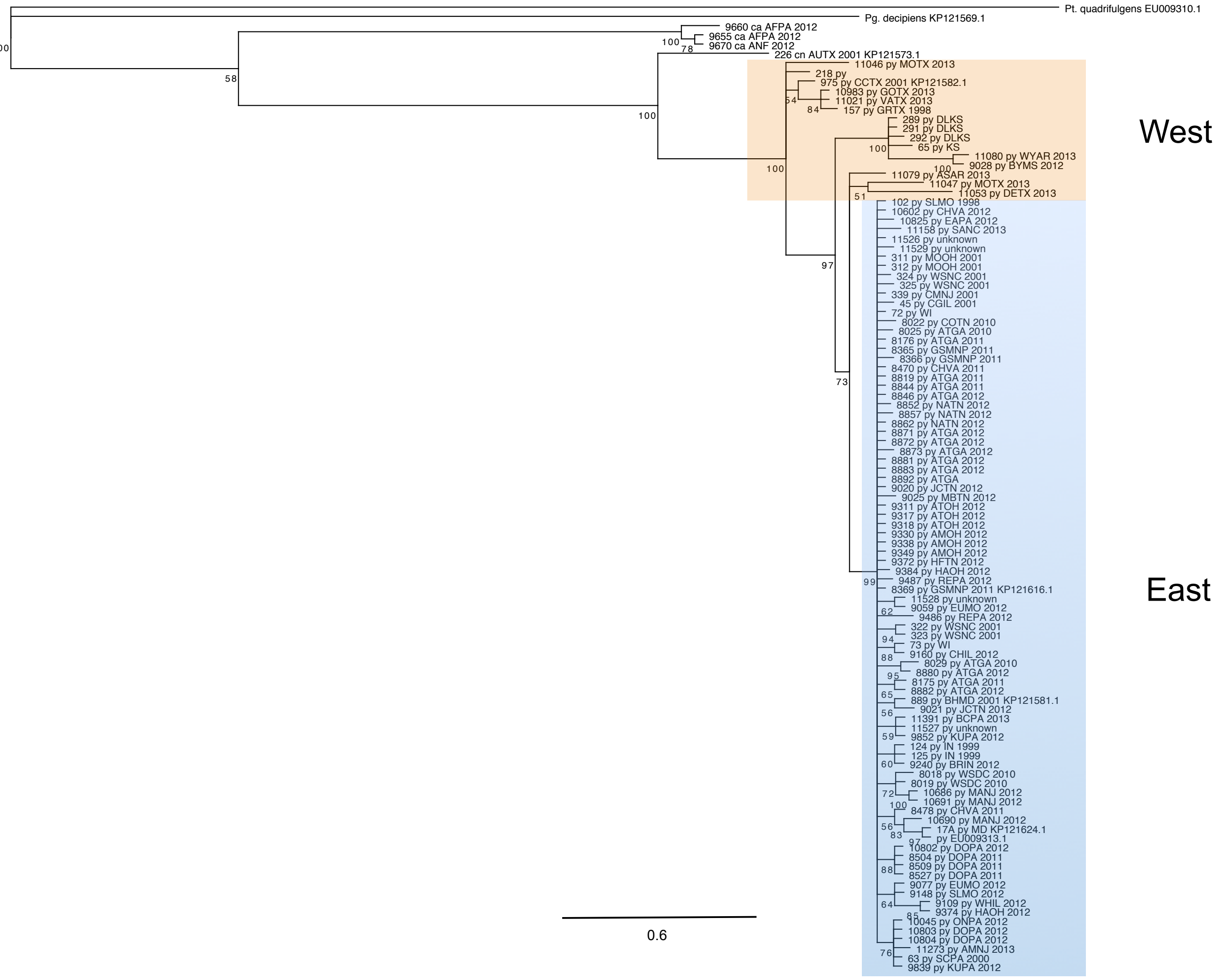

Supplemental Information: Note 3. Fst outlier analysis

Figure S3. Consensus set of Fst outliers without signaling loci

RAD locus-specific heterozygosity among populations versus Fst estimated using the FDIST (Beaumont & Nichols 1996) method implemented in LOSITAN (Antao *et al.* 2008). Values were averaged across 10 runs. Colors indicate candidate selection status based on strict consensus of all 10 LOSITAN runs and 2 Bayescan (Foll & Gaggiotti 2008) runs. Red = diversifying (N:29), gray = neutral (N:1977), blue = balancing/purifying (N:13).

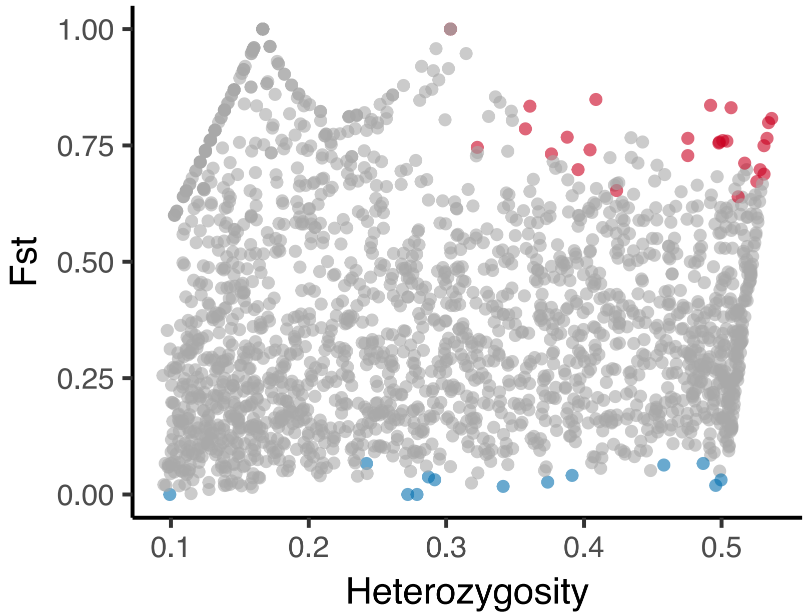

Figure S4. Consensus set of Fst outliers with signaling loci

RAD and signal locus-specific heterozygosity among populations versus Fst estimated using the FDIST (Beaumont & Nichols 1996) method implemented in LOSITAN (Antao *et al.* 2008). Values were averaged across 10 runs. Colors indicate candidate selection status based on strict consensus of all 10 LOSITAN runs and 2 Bayescan (Foll & Gaggiotti 2008) runs. Red = diversifying (N:33), gray = neutral (N:2058), blue = balancing/purifying (N:13). Circles = RAD loci, diamonds = adult luciferase, upward triangle = LW opsin, downward triangle = UV opsin.

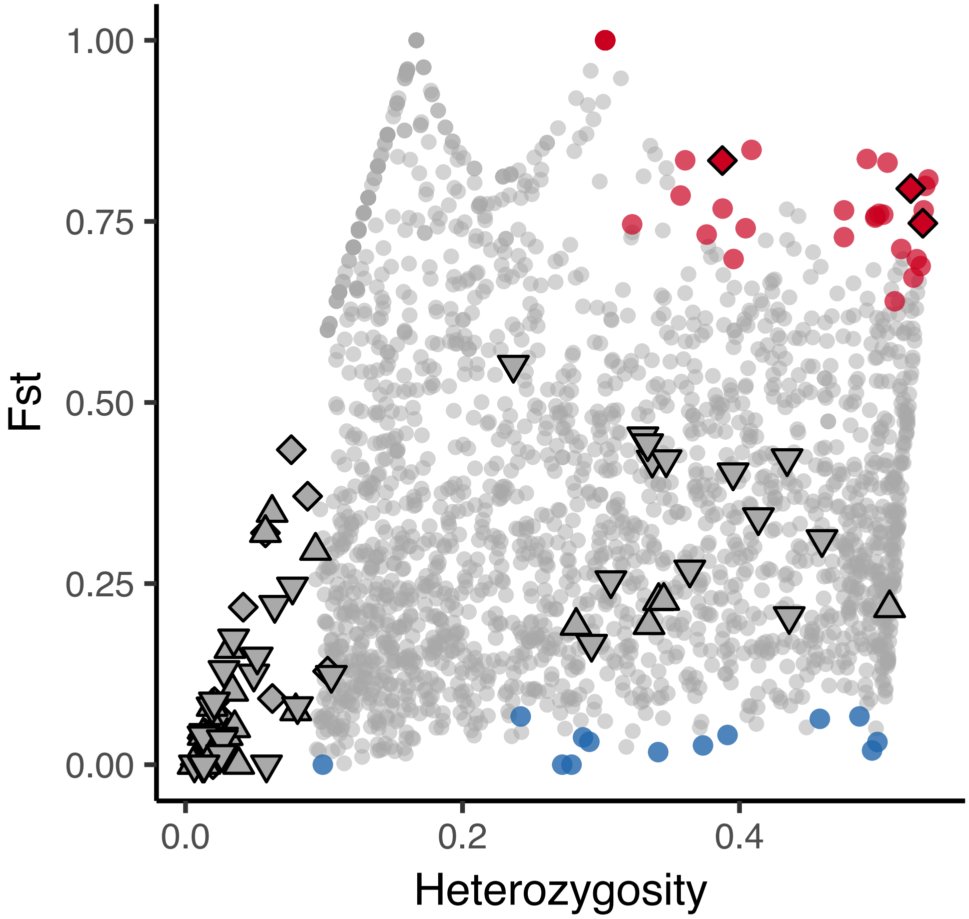

Figure S5. Neighbor-joining phylogenies of selected loci

Neighbor-joining trees were constructed using a conservative subset of the data (468 RAD SNPs with no missing data across individuals). Using LOSITAN, we identified loci under selection. We then divided the data into 3 sets: loci with evidence for (a) divergent selection, (b) balancing/purifying selection, and (c) neutral evolution are created neighbor-joining phylogenies for each set. The divergent loci tree shows a very narrow and stretched pattern, while the balancing loci tree shows a star pattern as expected. The tree constructed from neutral loci, despite being created from fewer SNPs overall, shows the same pattern as the tree constructed from 1977 loci, including those with missing data, as displayed in Figure 4 of the main text). Panel A shows the color key to the populations.

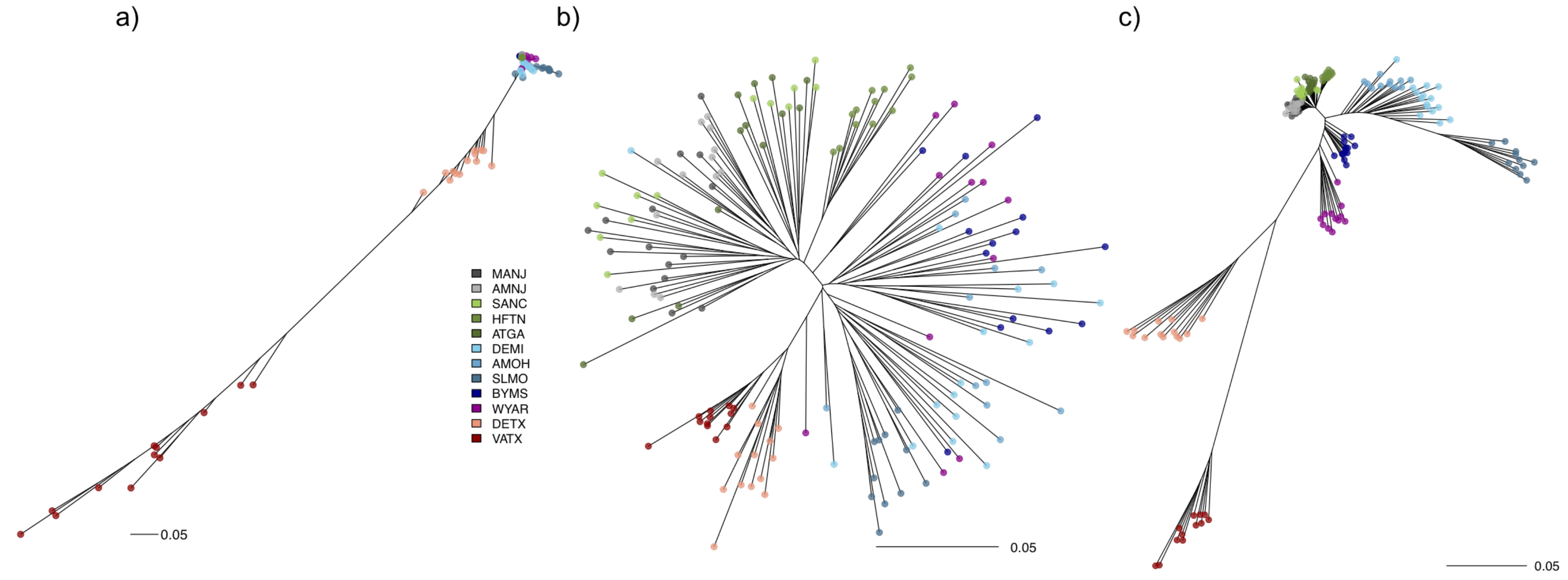

Supplemental Information: Note 4. Gene flow among populations

Figure S6. Pairwise Fst among populations

Visualization of pairwise Fst among populations as estimated from 1977 neutral loci. Populations are color coded according to Figure 4 (main text). Line width corresponds to Fst (wider/darker = higher gene flow; minimum = 0.01 between AMNJ and MANJ; maximum = 0.69 between VATX and HFTN).

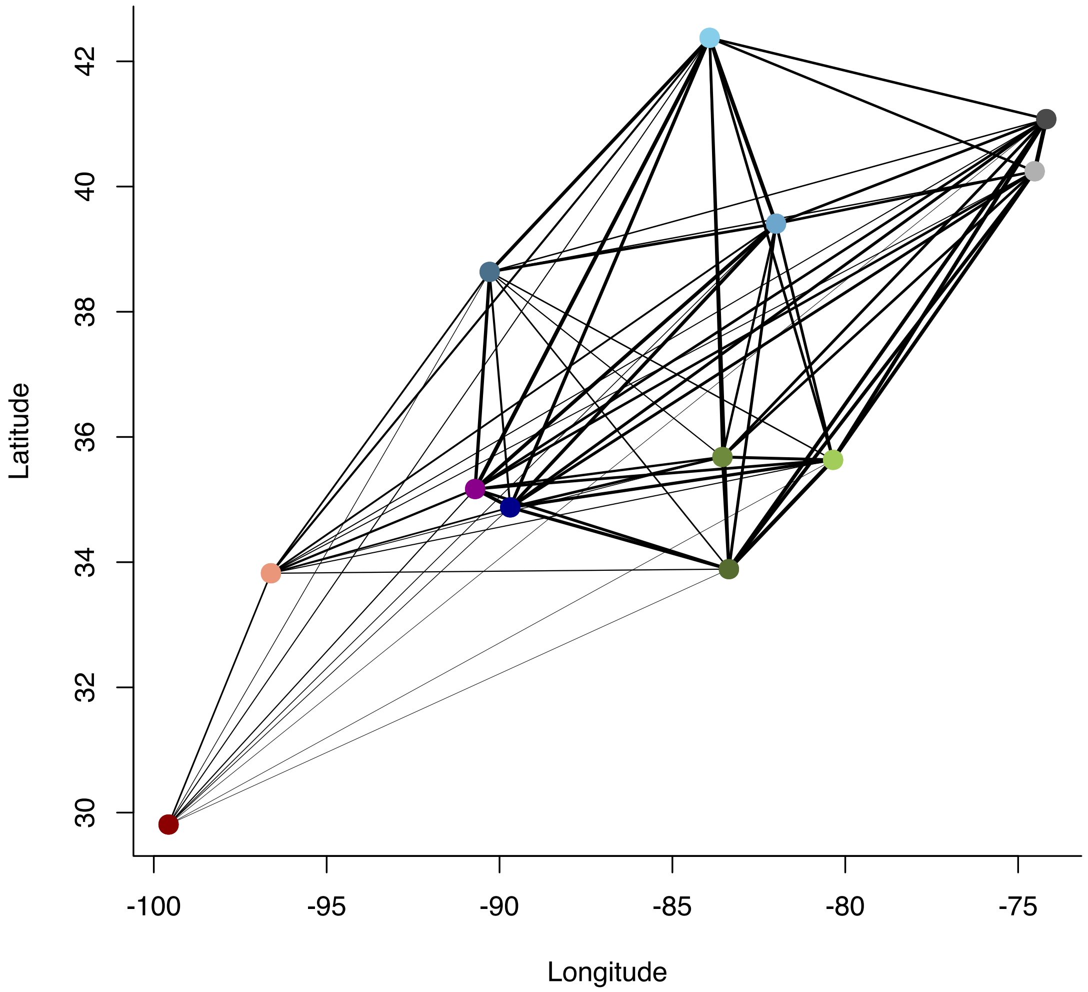

Table S4. Fst bootstrap results

Population1	Population2	Lower bound CI limit	Upper bound CI limit	p-value	Fst
ATGA	BYMS	0.150722703	0.180831144	0	0.164006463
ATGA	SLMO	0.429162953	0.461592083	0	0.444897321
ATGA	AMOH	0.215995152	0.243938001	0	0.231607016
ATGA	MANJ	0.080382549	0.105302481	0	0.09135951
ATGA	VATX	0.634176603	0.658362042	0	0.647182185
ATGA	DETX	0.48754632	0.521768328	0	0.507225201
ATGA	WYAR	0.221281068	0.250445622	0	0.2340924
ATGA	SANC	0.035792642	0.050920496	0	0.043395956
ATGA	AMNJ	0.102736709	0.129536309	0	0.11608779
ATGA	DEMI	0.243628403	0.26895501	0	0.257897858
ATGA	HFTN	0.174056291	0.208526498	0	0.193194795
BYMS	SLMO	0.34305066	0.37266733	0	0.360358866
BYMS	AMOH	0.121186687	0.142567705	0	0.132830879
BYMS	MANJ	0.196701242	0.227880786	0	0.211427726
BYMS	VATX	0.56265265	0.587173243	0	0.576639811
BYMS	DETX	0.40672598	0.439911621	0	0.424047653
BYMS	WYAR	0.112581936	0.131051271	0	0.121515355
BYMS	SANC	0.173701536	0.202569454	0	0.186657837
BYMS	AMNJ	0.208453804	0.240976498	0	0.223326257
BYMS	DEMI	0.144407301	0.168190052	0	0.155823371
BYMS	HFTN	0.254527213	0.284796912	0	0.268222546
SLMO	AMOH	0.235104054	0.265346511	0	0.250072234
SLMO	MANJ	0.447936591	0.47728936	0	0.464657725
SLMO	VATX	0.561088618	0.590451651	0	0.579142268
SLMO	DETX	0.38001151	0.41779692	0	0.401908124
SLMO	WYAR	0.148751547	0.175528253	0	0.161883031
SLMO	SANC	0.442876756	0.471410763	0	0.456595647
SLMO	AMNJ	0.455028246	0.488925611	0	0.473815126
SLMO	DEMI	0.132564366	0.152289528	0	0.142396724
SLMO	HFTN	0.488778197	0.517377537	0	0.502307609
AMOH	MANJ	0.242131185	0.274555654	0	0.259409923
AMOH	VATX	0.550128661	0.575468849	0	0.562667418
AMOH	DETX	0.372866733	0.408378421	0	0.393207841
AMOH	WYAR	0.092096491	0.112622534	0	0.101490278
AMOH	SANC	0.236548754	0.262012584	0	0.249918653
AMOH	AMNJ	0.254433427	0.286159893	0	0.272489505
AMOH	DEMI	0.059923533	0.074508787	0	0.066924429
AMOH	HFTN	0.299390099	0.327797781	0	0.313049819

MANJ	VATX	0.647852766	0.672227135	0	0.661445311
MANJ	DETX	0.507974281	0.536978118	0	0.522930476
MANJ	WYAR	0.247416304	0.280503967	0	0.265202982
MANJ	SANC	0.068489899	0.087139339	0	0.077587409
MANJ	AMNJ	0.007152468	0.016999262	0	0.011949562
MANJ	DEMI	0.268294742	0.29692681	0	0.282512789
MANJ	HFTN	0.220268423	0.261314804	0	0.240554257
VATX	DETX	0.408108643	0.450918798	0	0.430997373
VATX	WYAR	0.497059541	0.523207909	0	0.511350554
VATX	SANC	0.646722404	0.668297978	0	0.658477361
VATX	AMNJ	0.661148041	0.683133495	0	0.673455263
VATX	DEMI	0.505782833	0.536368755	0	0.521429755
VATX	HFTN	0.675830169	0.703086295	0	0.691555607
DETX	WYAR	0.317264053	0.351597365	0	0.335096851
DETX	SANC	0.503296919	0.536637001	0	0.520575854
DETX	AMNJ	0.516717696	0.546614669	0	0.533414753
DETX	DEMI	0.337563031	0.369484788	0	0.354743338
DETX	HFTN	0.538847576	0.572378377	0	0.555827999
WYAR	SANC	0.236956277	0.265870716	0	0.251167216
WYAR	AMNJ	0.260689079	0.293642606	0	0.278020103
WYAR	DEMI	0.055379706	0.070151368	0	0.062389583
WYAR	HFTN	0.303170472	0.331712778	0	0.316448814
SANC	AMNJ	0.087291008	0.105051694	0	0.09623546
SANC	DEMI	0.256791882	0.281689803	0	0.269391908
SANC	HFTN	0.19730125	0.236876922	0	0.218298244
AMNJ	DEMI	0.278666639	0.306981157	0	0.291700065
AMNJ	HFTN	0.240672412	0.283785433	0	0.262410663
DEMI	HFTN	0.309246963	0.339903449	0	0.323352206

Supplemental Information: Note 5. Additional signaling locus SNPs

Figure S7. Candidate signaling SNPs under balancing/purifying selection
Two LW opsin SNPs (99, 1204) and two UV opsins SNPs (299, 741) with evidence for balancing/purifying selection across populations as estimated from the consensus of 10 LOSITAN runs. These loci were not identified with BayeScan.

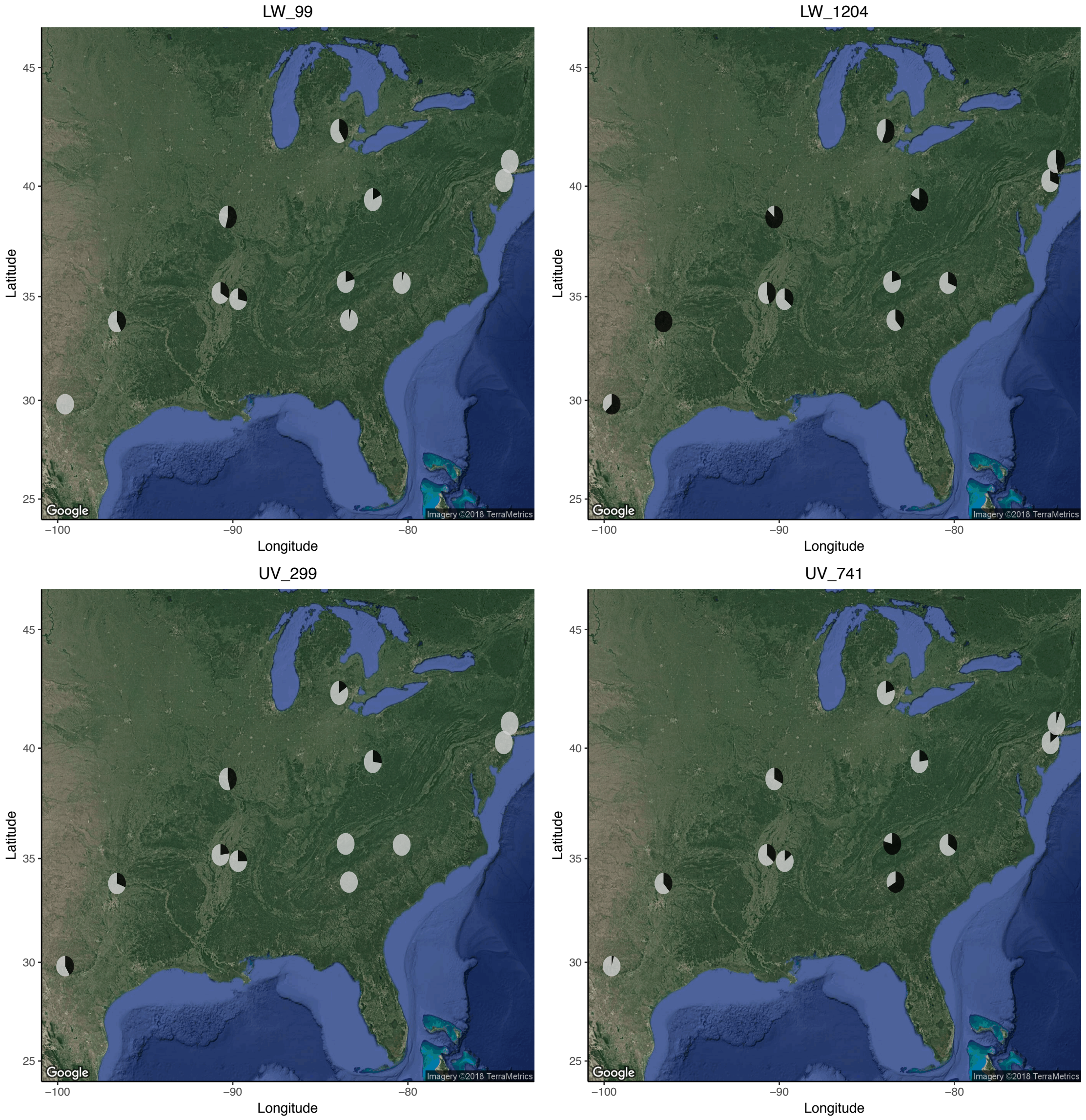

Supplemental Information: Note 6. Files on Figshare
(DOI: 10.6084/m9.figshare.5771979)

1.Specimens_used_for_sequencing
    Collection info for RADseq specimens
2a-c. Alignments used for assessing luciferase variation (.nex files)
3. lenient.hw.vcf
    vcf-format file for 2019 RAD loci used in analysis
4. lenient.hw.sigloci_noLUC804.gen.complete.txt
    genpop-format file of 2019 RAD loci and variable luciferase, LW opsin, and UV opsin SNPs
5.Selection_analysis_signal_loci.txt
    Tab-delimited file with results from 10 LOSITAN and 2 BayeScan runs
6. SUPPLEMENTARY FILE Optimization 7_8_17
    Word document showing stacks parameter optimization output

References

Antao T, Lopes A, Lopes RJ, Beja-Pereira A, Luikart G. LOSITAN: a workbench to detect molecular adaptation based on a Fst-outlier method. BMC Bioinformatics. 2008;9:323.

Beaumont MA, Nichols RA. Evaluating loci for use in the genetic analysis of population structure. Proc. Roy Soc. Lond. B. 1996;263:1619-1626.

Darriba D, Taboada GL, Doallo R, Posada D. jModelTest 2: more models, new heuristics and parallel computing. Nature Methods. 2012;9:772-772.

Edgar RC. MUSCLE: multiple sequence alignment with high accuracy and high throughput. Nucleic Acids Res. 2004;32:1792–7.

Faust L, de Cock R, Stanger-Hall K, Marion Z, Sander S. Allegheny National Forest June 2012 firefly survey: Forest and Warren Counties, PA. 2012.

Foll M, Gaggiotti O. A genome-scan method to identify selected loci appropriate for both dominant and codominant markers: a Bayesian perspective. Genetics. 2008;180:977–93.

Guindon S, Gascuel O. A simple, fast, and accurate algorithm to estimate large phylogenies by maximum likelihood. Sys Biol. 2003;52:696-704.

Hall DW, Sander SE, Pallansch JC, Stanger-Hall KF. The evolution of adult light emission color in North American fireflies. Evolution. 2016;70:2033–48.

Posada D. jModelTest: phylogenetic model averaging. Mol Biol Evol. 2008;25:1253-1256.

Ronquist F, Teslenko M, van der Mark P, Ayres DL, Darling A, Höhna S, *et al.* MrBayes 3.2: efficient Bayesian phylogenetic inference and model choice across a large model space. Sys Biol. 2012;61:539-542.

Sander SE, Hall DW. Variation in opsin genes correlates with signalling ecology in North American fireflies. Mol. Ecol. 2015;24:4679–96.

Stanger-Hall KF, Lloyd JE, Hillis DM. Phylogeny of North American fireflies (Coleoptera: Lampyridae): implications for the evolution of light signals. Mol. Phylogenet. Evol. 2007;45:33–49.

Stanger-Hall KF, Lloyd JE. Flash signal evolution in Photinus fireflies: character displacement and signal exploitation in a visual communication system. Evolution. 2015;69:666–82.
